# A Multiple Protease Strategy to Optimise the Shotgun Proteomics of Mature Medicinal Cannabis Buds

**DOI:** 10.3390/ijms20225630

**Published:** 2019-11-11

**Authors:** Delphine Vincent, Vilnis Ezernieks, Simone Rochfort, German Spangenberg

**Affiliations:** Agriculture Victoria Research, AgriBio, Centre for AgriBioscience, Bundoora, Victoria 3083, Australia; vilnis.ezernieks@agriculture.vic.gov.au (V.E.); simone.rochfort@agriculture.vic.gov.au (S.R.); german.spangenberg@agriculture.vic.gov.au (G.S.)

**Keywords:** protease digestion, trypsin/LysC, GluC, chymotrypsin, nLC-MS/MS, bovine serum albumin, BSA, bottom-up proteomics, middle-down proteomics, missed cleavage

## Abstract

Earlier this year we published a method article aimed at optimising protein extraction from mature buds of medicinal cannabis for trypsin-based shotgun proteomics (Vincent, D., et al. *Molecules* **2019**, *24*, 659). We then developed a top-down proteomics (TDP) method (Vincent, D., et al. *Proteomes* **2019**, *7*, 33). This follow-up study aims at optimising the digestion of medicinal cannabis proteins for identification purposes by bottom-up and middle-down proteomics (BUP and MDP). Four proteases, namely a mixture of trypsin/LysC, GluC, and chymotrypsin, which target different amino acids (AAs) and therefore are orthogonal and cleave proteins more or less frequently, were tested both on their own as well as sequentially or pooled, followed by nLC-MS/MS analyses of the peptide digests. Bovine serum albumin (BSA, 66 kDa) was used as a control of digestion efficiency. With this multiple protease strategy, BSA was reproducibly 97% sequenced, with peptides ranging from 0.7 to 6.4 kD containing 5 to 54 AA residues with 0 to 6 miscleavages. The proteome of mature apical buds from medicinal cannabis was explored more in depth with the identification of 27,123 peptides matching 494 unique accessions corresponding to 229 unique proteins from *Cannabis sativa* and close relatives, including 130 (57%) additional annotations when the list is compared to that of our previous BUP study (Vincent, D., et al. *Molecules* **2019**, *24,* 659). Almost half of the medicinal cannabis proteins were identified with 100% sequence coverage, with peptides composed of 7 to 91 AA residues with up to 9 miscleavages and ranging from 0.6 to 10 kDa, thus falling into the MDP domain. Many post-translational modifications (PTMs) were identified, such as oxidation, phosphorylations, and N-terminus acetylations. This method will pave the way for deeper proteome exploration of the reproductive organs of medicinal cannabis, and therefore for molecular phenotyping within breeding programs.

## 1. Introduction

The recent revised legislation on medicinal cannabis has triggered a surge of medical and clinical research studies evaluating the effect of major cannabis components on human health. *C. sativa* has been dubbed “the plant of the thousand and one molecules” [1] owing to its propensity to produce a plethora of phytochemicals with myriad of biological activities as well as fibrous components. Out of the 500 compounds that have been described thus far [2,3,4,5], more than 90 are phytocannabinoids, including cannabidiolic acid (CBDA) [6] and delta 9-tetrahydrocannabinolicacid (THCA) [7]. The biosynthetic pathway of *C. sativa* phytocannabinoids and the characterization of related enzymes was recently elucidated [1]. The main enzymes are 3,5,7-trioxododecanoyl-CoA synthase (OLS, a polyketide synthase) and olivetolic acid cyclase (OAC) acting in succession to convert hexanoyl-CoA into olivetolic acid (OLA). Geranylpyrophosphate:olivetolate geranyltransferase (GOT) catalyses the alkylation of OLA with geranyldiphosphate leading to the formation of cannabigerolic acid (CBGA). THCA synthase (THCAS) converts CBGA to THCA, while CBDA synthase (CBDAS) forms CBDA. Finally, CBCA synthase (CBCAS) produces cannabichromenic acid (CBCA).

Whilst several genome sequencing projects are underway [8,9,10], *C. sativa* proteome remains poorly characterized with only eleven reports published so far [11,12,13,14,15,16,17,18,19,20,21], compounded by the fact that only 509 *C. sativa* protein accessions (0.006%, out of 8,344,090 *viridiplantae* accessions) are currently available in the public reference protein database UniprotKB (October 2019, https://www.uniprot.org/uniprot/?query=taxonomy:3483%20taxonomy:%22Rosales%203744%22%20cannabis%20sativa). Early this year, we published results on bottom-up proteomics (BUP) demonstrating optimum protein extraction from *C. sativa* mature buds when an initial precipitation step was followed by resuspension into a guanidine-hydrochloride buffer [11]. Using a trypsin-based shotgun approach, we identified 5675 peptides matching 160 accessions from *C. sativa* and close relative species (hop and Chinese grass), including all the enzymes involved in the phytocannabinoid biosynthetic pathway. In this peptide-centric approach, protein coverage ranged from 1% (Photosystem I P700 chlorophyll a apoprotein A1, 83 kDa) to 72% (Photosystem I iron-sulfur center, 9 kDa) and post-translational modifications (PTMs) were underrepresented. For instance, the smallest of the phytocannabinoid enzymes, OAC (12 kDa) was identified with three unique peptides covering 34% of the AA sequence and no PTM was detected. BUP was advantageous as it allowed for the identification of cannabis proteins of low abundance and high MW (e.g., Protein Ycf2, 271 kDa). We then developed a top-down proteomics (TDP) strategy complementary to BUP which allowed the detection of unreported PTMs of the identified intact cannabis proteins, such as the excision of the N-terminus M, and the presence of methylations, acetylations, and phosphorylations [12]. We have demonstrated the complementarity of BUP and TDP not only in buds from medicinal cannabis [11,12] but also in cow’s milk [22,23,24,25].

Meyer and colleagues have stated the need to undertake high-throughput bottom-up strategies to determine which proteins are present in the species of interest; however, they exercise caution with respect to describing cell events without 100% protein sequence coverage [26]. BUP has become the core of MS-based proteome analysis propelled by the optimization of all the steps involved in a proteomics workflow including sample preparation, protein digestion, peptide separation by LC, fragmentation by MS, and database search algorithms [27,28]. The serine protease trypsin claims monopoly in BUP approaches. Trypsin is one of the most important digestive proteases of the vertebrates with the essential role of cleaving dietary proteins into peptides with a specificity for R and K residues (reviewed in [29]). Trypsin owes its top position in BUP to its low cost commercialisation, high efficiency, cleavage-site specificity, and production of tryptic peptides amenable to MS. Miscleavages have been reported as a result of the protease skipping a seemingly cleavable residue, typically when R or K is followed by a P [30]. Neighbouring negatively charged AA residues (E and D) and phosphorylated S or T also result in miscleavages [31,32]. This propensity must be accounted for in the search method by increasing the number of missed cleavage sites; this only taxes computing cycles without compromising the output [29]. Trypsin exhibits a somewhat lower cleavage efficiency towards K than R residues. This incomplete protein digestion can be alleviated by an additional digestion step with the lysyl endopeptidase LysC from *Lysobacter enzymogenesis* which cleaves at the carboxyl terminus of K residues and operates under the same conditions as trypsin (pH 7–9), thereby yielding fewer missed cleavages [33,34,35,36]. So efficient is this combination that the vendor Promega is now commercializing a ready-to-use trypsin/LysC mixture for shotgun proteomics experiments which we took advantage of in the present study.

Proteases other than trypsin are commercially available. Chymotrypsin preferably cleaves large hydrophobic residues such as Y, F and W and to a lesser extent L and M, and as such it is less specific than trypsin. Qualitatively and quantitatively speaking, chymotrypsin generates peptides which cover a proteome space orthogonal to that of trypsin [28]. The glutamyl peptidase I (GluC) from *Staphyloccous aureus* is a highly specific protease as it cleaves at the C-terminal side of E residues under acidic conditions (pH 4) whereas under alkaline conditions (pH 8) it additionally cleaves at the C-terminal side of D residues [37]. GluC therefore also acts orthogonally to trypsin and chymotrypsin. GluC cleaves proteins less frequently than chymotrypsin, and as a result, creates longer peptides which fill the gap between BUP and TDP so called middle-down proteomics (MDP). TDP typically explores intact proteins of 10–30 kDa, whereas trypsin-based BUP generally yields short peptides from 0.7–3 kDa. At the interface, MDP strives to yield fewer and larger polypeptides spanning 3–10 kDa [38]. MDP is particularly advantageous as the complexity of the digests diminishes while proteome coverage deepens, offering the identification of splice-variants and other isoforms as well as PTMs. The shorter the proteolytic peptides, the more difficult the protein inference, i.e., assembling the sequences of identified peptides into proteins [39]. MDP thus delivers an elegant solution to BUP shortcomings, yet it remains to be wholeheartedly embraced by the proteomics community as evidenced by the paucity of publications on complex biological matrix thus far. Examples of thorough characterization proteoforms by MDP include histones [40,41,42] and monoclonal antibody [43]. 

As was exemplified with the successful combination of trypsin with LysC, the limitations of one protease can be compensated for with one or more proteases exhibiting orthogonal specificities when applied in parallel or in series on the same sample. Vandermarliere and colleagues performed an in-silico cost-benefit analysis of using several proteases on their own or in combination on the human proteome and found that parallel digests are indeed beneficial, augmenting both the sequence coverage and the number of identified proteins [29]. Such multiple protease strategies have been successfully adopted by the proteomics community, particularly to detect PTMs and process tissues whose protein cohorts are less prone to trypsin digestion. Some groups have adopted low specificity proteases such as elastase, subtilisin, proteinase K, and thermolysin [44,45,46]. Membrane proteins typically lack tryptic cleavage sites and harbor numerous hydrophobic AAs which are more amenable to digestion with chymotrypsin. Chymotrypsin has successfully been used in combination with trypsin or other proteases to delve deeper into the membrane proteomes [47,48,49]. Other groups have used highly specific proteases such as GluC, LysC, ArgC and AspN to dig deeper into the proteomes of complex biological samples, in particular using human body fluids [50,51] and HeLa cell lysates [38,52]. Generating longer peptides successfully improved the detection and sequence coverage of such complex proteomes in an MDP approach. While a combination of three proteases was often used, some groups have increased the number of proteases combined together [53,54,55]. For instance, Swaney and colleagues evaluated the use of five proteases (LysC, GluC, ArgC, AspN, and trypsin) to improve the ability to comprehensively characterize entire proteomes of yeast, and in particular detect phosphorylation sites [55]. Guo and colleagues have employed seven proteases of low specificity (chymotrypsin and elastase), medium specificity (trypsin) and high specificity (GluC, LysC, AspN, and ArgC) in a complex combinatorial design of single-, double-, and triple-enzyme digests to devise the optimum sample digestion and proteome coverage of HeLa cell lysates [54]. While they also report an increase in both protein identifications and mean sequence coverage, they further conclude that dynamic range rather than enzyme bias is the most limiting factor to proteome exploration. 

Fulfilling BUP and MDP potentials through the use of multiple proteases of various specificity ensures both a higher coverage of AA sequences and deeper proteome exploration, which is critical to discriminate closely related protein isoforms and detect various PTM sites, as well as robust and precise protein identification and quantification. Based on our previous BUP experience in which protein recovery from mature cannabis buds was optimised [11], the present study aims at improving our shotgun proteomics workflow, in particular the digestion steps, to identify more proteins with greater confidence and discover more PTMs. To this end, we developed a combinatorial multiple protease method on mature buds from medicinal cannabis. We chose three orthogonal digestions, namely chymotrypsin (of low specificity targeting hydrophobic residues Y, F, W, and to a lesser extent L), a ready-to-use mixture of trypsin/LysC (of medium specificity targeting positively charged residues R and K), and GluC (high specificity targeting negatively charged residues mostly E and occasionally D under our experimental conditions). These enzymes were carefully selected based on their specificity to yield peptides spanning from 0.5 to 10 kDa as to cover both BUP and MDP ranges. We first tested the digestion efficiency of the proteases on their own or in sequence using the shotgun reference protein BSA, and then applied the method to complex plant samples. Single-, double- and triple-enzyme digests were analysed by nLC-MS/MS. The results are discussed in terms of reproducibility, number of identified peptides, missed cleavages, PTM detection, AA sequence and proteome coverages.

## 2. Results and Discussion

**Figure 1:**
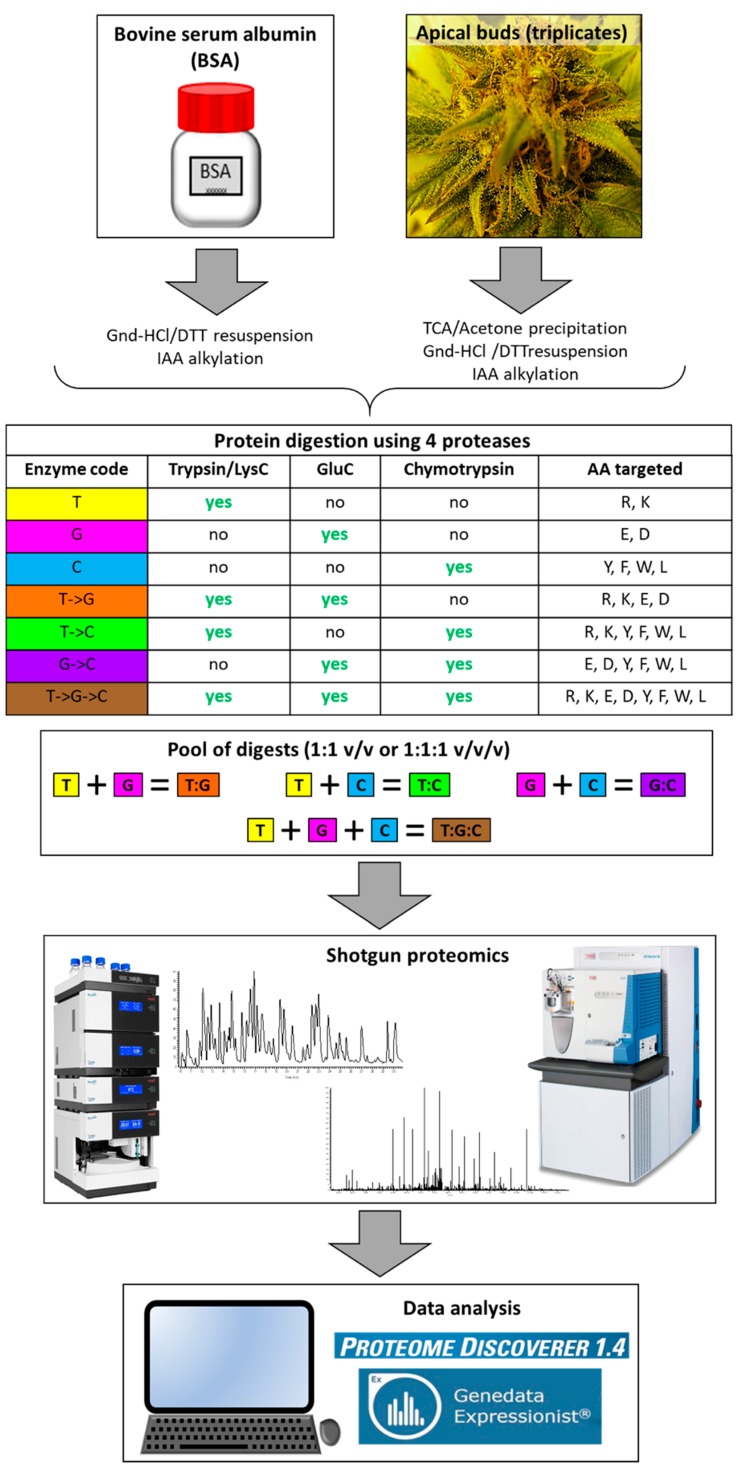
Experimental design.

In a previous study, we applied the gold standard protease in proteomics, trypsin, to digest cannabis proteins and analysed them using a BUP approach [11]. The proteome of mature apical buds was satisfactorily covered as assessed by the identification of all the enzymes involved in the biosynthesis of phytocannabinoids, along with many other enzymes from cannabis secondary metabolism (e.g., isoprenoids, terpenoid, and phenylpropanoid). However, AA sequence coverage never reached 100% and only a handful of PTMs were identified. More proteins identified based on more proteotypic peptides to ensure deeper proteome coverage can be reached by adopting a multiple protease strategy as demonstrated by a wide body of evidence [29,38,44,45,46,47,48,50,51,52,53,54,55]. In this experiment, a trypsin/LysC (T) mixture, GluC (G) and chymotrypsin (C) were applied on their own or in combination, either subsequentially (denoted by an arrow “->”) in a serial digestion fashion, or by pooling individual digests together (denoted by a colon “:”). The analytical method was first tested on BSA and then applied to complex plant samples. The experimental design is schematised in Figure 1. 

### 2.1. Shotgun Proteomics on the Test Protein BSA

BSA is used as a positive control in our experiment as it is the gold standard used for shotgun proteomics [56]. It is a 66 kDa monomeric protein particularly amenable to trypsin digestion. The sequence coverage of BSA tryptic digest is often used to evaluate instrument performance as it is sensitive to both MS and MS/MS method settings. We routinely use BSA as a quality control of both enzymatic digestion and nLC-MS/MS analyses [11,24]. 

#### 2.1.1. LC-MS/MS Data from BSA Digests Are Very Reproducible

Each BSA digests underwent nLC-MS/MS analyses in which each duty cycle comprised a full MS scan was followed by CID MS/MS events of the 20 most abundant parent ions above a 10,000 counts threshold. Supplementary Figure S1 displays the nLC-MS profiles corresponding to one replicate of each BSA digest. The peptides elute from 9 to 39 min corresponding to 9–39% ACN gradient, respectively and span *m*/*z* values from 300 to 1600. Visually, LC-MS patterns from samples subject to digestion with GluC followed by chymotrypsin (G->C) are relatively less complex than the other digests.

Technical duplicates of the BSA digests yield MS and MS/MS spectra of high reproducibility as can be seen in Table 1. 

All LC-MS patterns are highly complex with a multitude of ions. The number of MS peaks vary from 77,085 (G->C rep 1) to 100,001 (G:C rep 2) across all patterns and SDs range from 440 (T) to 3794 (T->C) with coefficient of variations (%CVs) always lower than 5%, even though a full set of eleven digest combinations (Figure 1) was run first (technical replicate 1), and then fully repeated in the same order (technical repiclate 2) with no randomization applied. The number of MS/MS events ranges from 5163 (6%, G->C rep 2) to 11,311 (13% T->G rep 1), which amounts to 10% of all the MS peaks on average (Table 1). The number of MS/MS events per sample is determined by the duration of the run (50 min) and the duty cycle (3 s) which in turn is controlled by the resolution (60,000), number of microscans (2) and number of MS/MS events per cycle (20). In our experiment, a 50 min run allows for 1,000 cycles and 20,000 MS/MS events. Proteotypic peptides elute for 30 min, thus allowing for a maximum of 12,000 MS/MS scans. With an average number of 9297 MS/MS spectra obtained (Table 1), 77% of the potential is thus achieved. Duty cycles can be shortened by lowering the resolving power of the instrument, minimizing the number of microscans and diminishing the number of MS/MS events. The MS/MS data were searched against a database containing the BSA sequence using SEQUEST algorithm for protein identification purpose. Of all the MS/MS spectra generated in this study, between 475 (9%, G->C rep 2) and 2428 (24%, T:C rep 1) are successfully annotated as BSA peptides (Table 1). On average, 17% of the MS/MS spectra yield positive database hits, which amounts to an average of 1.8% of MS peaks. The list of BSA peptides identified in this study is available in Supplementary Table S1. Trypsin/LysC yields 68 unique BSA peptides, GluC yields 79 unique BSA peptides, and chymotrypsin yields 104 unique BSA peptides. These values are greater than those reported by Giansanti and colleagues, with 37 trypic peptides, 31 GluC-associated peptides and 24 chymotryptic peptides [53], which explains our higher AA sequence coverage (discussed below). In a previous BUP study, BSA was identified with 40 unique peptides obtained using tryspin on its own [24], therefore the mixture trypsin/LysC enhances the digestion of BSA. The percentages of Table 1 are turned into a histogram in Supplementary Figure S2 to better visualize the proportions of meaningful data across proteases. The proportion of MS peaks fragmented by MS/MS remains constant across BSA digests, oscillating around 10 ± 3% (light grey bars). The proportions of MS/MS spectra annotated in SEQUEST (i.e., successful hits) however show more variation across proteases (black bars). Higher percentages are reached when trypsin/LysC is employed on its own or in combination with GluC and/or chymotrypsin (Supplementary Figure S2). This is expected as BSA is notoriously amenable to trypsin digestion and often used as shotgun proteomics standard [56]. 

BSA (P02769) mature primary sequence contains 583 AAs, from position 25 to 607; the signal peptide (position 1 to 18) and propeptide (position 19 to 24) are excised during processing. In theory, BSA should favorably respond to each protease as it contains plethora of the AAs targeted during the digestion step. Supplementary Figure S3A indicates the AA composition of BSA. Targets of chymotrypsin (L, F, Y, and W) account for 19% of BSA sequence, targets of GluC (E and D) represent 17% of the sequence, and targets of trypsin/LysC (K, R) make 14% of the total AA composition of BSA. As these percentages are comparable, the difference in the numbers of MS/MS spectra successfully matched by SEQUEST from one protease to another cannot be attributed to digestion site predominance. When we compare these predicted percentages to those observed in our study based on unique peptides (Supplementary Figure S3B), we can see that all the targeted AAs indeed undergo cleavage. The predicted rate always exceeds the observed one, but only moderately for W, Y, E, K, and R residues (less than 1.5% difference). However, F, L, and in particular D residues present an observed cleavage rate that is much lower than the predicted one (Supplementary Figure S3B). The higher specificity of GluC towards E is explained by the use of ammonium bicarbonate as diluent. The lower cleavage efficiency towards L and D, among the most abundant AAs in BSA (Supplementary Figure S3A), is likely to impact sequencing efficiency.

If we report the number of successfully annotated MS/MS events to that of MS peaks, percentages fluctuated from 1.0% (G->C) to 2.6% (T:C) (Table 1 and dark grey bars in Supplementary Figure S2). This suggests that some of the digestion conditions devised in this work are better suited to sequence BSA than others. This also highlights that the vast majority of the ions generated by nLC-MS remain anonymous because either they are not selected as precursors or despite their being fragmented, the MS/MS output is not recognized in the searched database. Some of this is explained by the fact that the BSA standard used in their study is 98% pure and contains other proteins [23,24] that are ignored during the search stage on purpose. Maximizing the proportion of ions chosen for MS/MS fragmentation should improve database hit rates.

#### 2.1.2. Each Protease on Their Own or Combined Yield High Sequence Coverage of BSA

**Figure 2:**
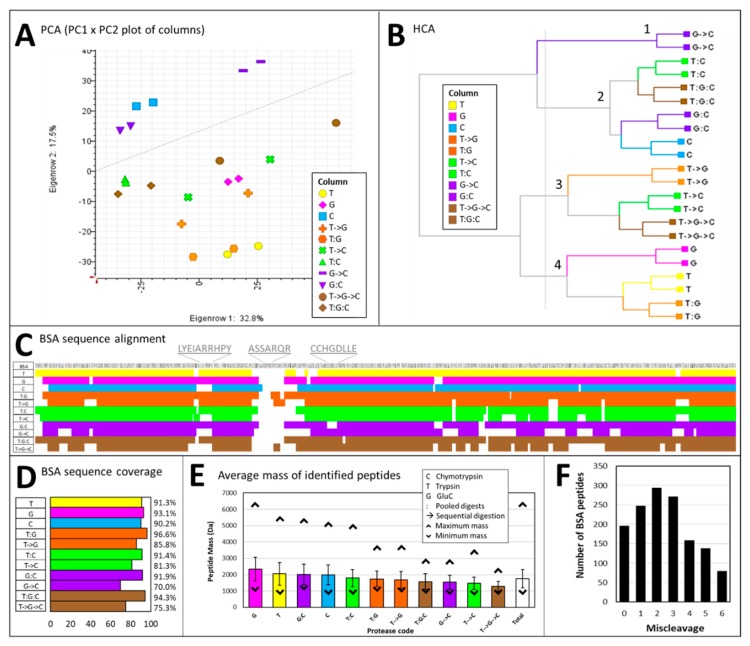
Protease digestion tests on bovine serum albumin (BSA). (**A**) Principal component analysis (PCA) of the BSA identified peptides. (**B**) Hierarchical clustering analysis (HCA) of the BSA identified peptides. (**C**). Identified peptides aligned onto BSA AA sequence of the mature protein. (**D**). BSA sequence coverage achieved using the various proteases on their own or in combination. (**E**) Average mass (Da) of BSA peptides resulting from the three proteases acting on their own, sequentially, or pooled; vertical bars denote standard deviation (SD). (**F**) Distribution of number of identified peptides according to the number of miscleaveages. Downward arrowhead (v) denotes the minimum peptide mass and upward arrowhead (^) denotes the maximum peptide mass.

Figure 2 summarises the statistical tests performed and the BSA sequence information in addition to a visual assessment of BSA sequencing success for each combination of proteases.

Principal component analysis (PCA) shows that technical duplicates group together (Figure 2A). BSA samples arising from enzymatic digestion using chymotrypsin (C) in combination or not with GluC (G->C and G:C) separate from the rest, particularly tryptic digests (T), along PC 2 explaining 17.5% of the variance. Hierarchical clustering analysis (HCA) confirms PCA results and further indicates that samples treated with trypsin/LysC (T) and GluC (G) on their own or pooled (T:C) form one cluster (cluster 4, Figure 2B). The closest cluster (cluster 3) comprises all the samples subject to sequential digestions (represented by an arrow ->), except for digests resulting from the consecutive actions of GluC and chymotrypsin (G->C) which constitute a cluster on their own (cluster 1). The last cluster (cluster 2) groups chymotryptic samples with the remaining pooled digests (represented by a colon). The fact that clusters 1-3 contains samples treated with chymotrypsin (except for T->G) suggests that this protease produces peptides with unique properties which impact the down-stream analytical process. This confirms that chymotrypsin indeed acts in an orthogonal fashion to trypsin as previously noted [28].

Based on the 589 unique BSA peptides identified in this study (Supplementary Table S1), we generated a BSA sequence alignment map (Figure 2C) and coverage histogram (Figure 2D). All digests considered, BSA sequence is at least 70% covered (G->C), up to 97% (T:G) (Figure 2D), with an average of 87% coverage. Despite this almost complete coverage, the seven AA-long area positioned between residues 214 and 220 (ASSARQR) resists digestion, even though R residues targeted by trypsin are present (Figure 2C). Perhaps BSA denaturation is incomplete under our experimental conditions and this domain is not exposed to the proteases. When we look at each type of digest individually, other areas resisting cleavage emerge, some of them common across different digests (e.g., position 162-171, LYEIARRHPY, shared between C, T->C, G->C, and T->G->C) or unique to a particular digest (e.g., position 268 to 275, CCHGDLLE, in G:C) (Figure 2C). If we compare digests obtained using a unique enzyme, excellent BSA sequence coverage are observed: 91.3% for trypsin/LysC, 93.1% for GluC, and 90.2% for chymotrypsin (Figure 2D). This also demonstrates comparable digestion efficiencies under our experimental conditions which abode to the protease manufacturer’s guidelines. In a previous BUP study on milk, digestion of BSA using trypsin on its own achieved 65% AA sequence coverage [24]. Therefore, greater confidence in protein assignment is achieved by combining LysC to trypsin, as was reported [33,34,35,36]; this could also arise from a more optimum usage of the instrument duty cycle. In the protocol standardized by Giansanti and colleagues, BSA sequence coverage varied significantly depending on the protease used, with trypsin achieving 78.4%, GluC 61.9% and chymotrypsin 57.8% [53]. From our results, we conclude that, while trypsin is usually the gold standard for shotgun proteomics, these alternative enzymes should also be considered. 

If we now turn our attention to digests obtained using multiple enzymes and compare sequential digestions (->) with pooled digests (:), we observe better alignment and coverage when individual digests are combined than when proteases are added. For instance, T->C digests covers 81% of the BSA sequence while T:C digest reach 91% coverage (Figure 2D); the 10% difference represents 56 AAs. This is better exemplified when the three proteases are used together, with a 75% coverage in T->G->C samples and 94% coverage in T:G:C samples (Figure 2D); the 19% difference represents 111 AAs. A similar multiprotease method was exploited by Liang and colleagues to achieve high sequence coverage of the toxic protein ricin and distinguish it from its close relative RCA120 agglutinin [57]. Irrespective of the proteases used, resorting to different enzymes to digests proteins from a complex biological matrix has proven extremely beneficial over the years as this strategy augments the AA sequence coverage and therefore strengthens protein inference as attested by the numerous publications on this topic [38,44,45,46,47,48,50,51,52,54,55].

The masses of identified BSA peptides ranged from 688 to 6412 Da, with an average of 1,758 ± 753 Da (Figure 2E), spanning 5 to 54 AA residues. GluC is the enzyme that generates the longest peptides with an average of 2342 ± 1052 Da, followed by trypsin/LysC (2053 ± 1000 Da), the mixture GluC/chymotrypsin (G:C, 2008 ± 765), and chymotrypsin (1989 ± 901 Da). As demonstrated elsewhere [38,40,41,42], GluC on its own produces peptides large enough to undertake MDP analyses. The smallest peptides result from the sequential actions of GluC and chymotrypsin (G->C, 1541 ± 511 Da), trypsin/LysC and chymotrypsin (T->C, 1481 ± 567 Da), and all three proteases (T->G->C, 1295 ± 348 Da). This confirms that adding multiple proteases to a sample enhances protein cleavage or gives rise to additional novel peptides. 

#### 2.1.3. The Number of Miscleavages Is a Critical Parameter in Database Search

BSA peptides contain up to six miscleavages, with the majority (59%) presenting 1-3 miscleavages (Figure 2F). The different digestion conditions peak at different miscleavages as can be seen in Supplementary Figure S4. For instance, the greatest number of tryptic and chymotryptic peptides exhibit one miscleavage while GluC-released peptides containing three miscleavages are the most numerous. The longest peptide (VSRSLGKVGTRCCTKPESERMPCTEDYLSLILNRLCVLHEKTPVSEKVTKCCTE, 6.4 kDa) released from the action of GluC contains eight charges and six miscleavages; it has a SpScore of 1572 and a Xcorr of 4.14 (Supplementary Table S1). 

Traditionally in shotgun proteomics where trypsin is used to perform the enzymatic digestion of the protein extracts, the maximum number of missed cleavages is set to two. In our study, a significant proportion of BSA peptides (47%) contain more than two miscleavages (35% of BSA tryptic peptides) therefore we would have ignored almost half of the hits which such a restraint. Setting the number of allowed miscleavage to zero gives maximum discrimination between correct and incorrect matches but also assumes that the digestion was complete while increasing this number assumes digestion is partial [58]. Setting too high a number can be computationally taxing as it significantly augments the number of calculated peptide masses to be matched against the experimental data. In our experiment, we purposefully did not set a limit on the number of missed cleavages to assess whether longer peptides could be identified this way, as indeed is the case. In an MDP study on cell lysates, up to four missed cleavages were set for GluC in the search method [38]. Likewise, up to four miscleavages are recommended for chymotrypsin and GluC, and only two for trypsin [53]. 

For a given protein accession, the excess of limit-digested peptides (ELDP) is computed by subtracting the number of matched peptides with a missed cleavage site from the number of matched peptides without miscleavage [59]. ELDP is used to assess the completeness of BSA digestion. As the number of BSA peptides with one (248, Figure 2F) or more (941) miscleavages far exceeds the number of BSA peptides that bear no miscleavage (196), we can conclude that BSA digestion was partial and therefore the number of missed cleavage sites had to be increased. 

Even though our experimental design cannot determine which missed cleavage parameters should have been used and that one might argue that too many missed cleavages are allowed, we have confidence in our results for the following reasons: (1) the decoy database search eliminates false positives, (2) only the peptides displaying a “high” minimum confidence in Proteome Discover (i.e., minimal FDR score for 2+ charge state = 0.01) are kept; indeed very long peptides displaying numerous miscleavages present very high score (Supplementary Table S1), (3) while up to ten miscleavages are permitted, a maximum of six are detected in the BSA dataset (Figure 2F); if false positives were in the dataset, the whole range of miscleavages (i.e., up to 10) would have been detected. The reasons why the best studied protease, trypsin, regularly misses cleavage sites have been kinetically explained [29,32]; the propensity of GluC and chymotrypsin to miss seemingly cleavable sites should also be mechanistically elucidated. In the meantime, we recommend applying a number of missed cleavages greater than two during the database search stage.

From this experiment, we can conclude that BSA is highly amenable to enzymatic digestion by trypsin/LysC, GluC and chymotrypsin. Pooling the individual digests does not affect the nLC-MS/MS analysis as attested by the high sequencing coverage. Using multiple proteases consecutively yields relatively lower sequence coverage of BSA. This study also benchmarks the use of these digestion enzymes in our laboratory which can then be applied to complex biological samples such as plant protein extracts from mature buds of medicinal cannabis.

### 2.2. Medicinal Cannabis

Medicinal cannabis mature apical bud samples were extracted in triplicate from three plants of the same cultivar and aliquoted into equal protein content prior to performing the various enzymatic digestions in duplicate as schematized in Figure 1. 

#### 2.2.1. LC-MS Patterns from Medicinal Cannabis Digests Are Very Complex

As expected with protein-rich samples, the LC-MS patterns are very complex with cannabis peptides eluting from 9–39 min (9–39% ACN gradient) exhibiting *m*/*z* values spanning from 300 to 1700 (Supplementary Figure S5), which is comparable to what we previously observed [11]. The nLC-MS patterns corresponding to digests resulting from the action of GluC and chymotrypsin either sequentially (G->C) or mixed (G:C) present less peaks (Supplementary Figure S5). Digests resulting from the consecutive action of all three proteases (T->G->C) were consistently troublesome with the nanospray becoming sporadic from time to time, for no obvious reason. Furthermore, the pooled digests (denoted with a colon symbol) yielded unreproducible LC-MS patterns due to unstable nanospray (data not shown) therefore we did not include them to the rest of the analysis. 

**Figure 3:**
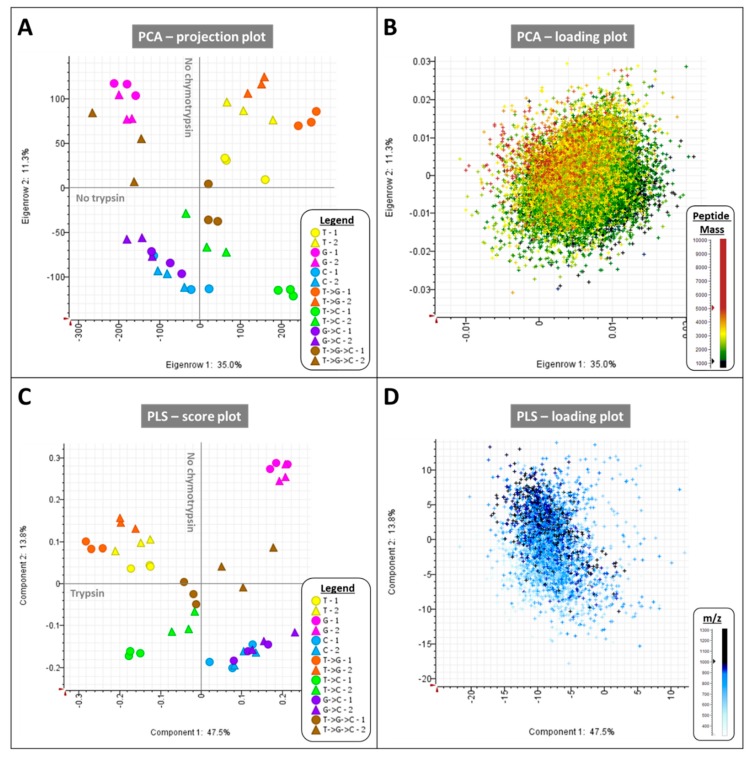
Principal component analysis (PCA) and partial least square (PLS) analysis of medicinal cannabis digests. (**A**) PCA projection plot of PC1xPC2 featuring the 42 digest samples resulting from the action of one protease (T, G, or C), two (T->G, T->C, or G->C), or three proteases (T->G->C) applied sequentially; (**B**) PCA loading plot of PC1xPC2 featuring the 27,635 *C. sativa* peptides identified and coloured according to their deconvoluted masses. (**C**) PLS score plot of LV1xLV2 featuring the 42 digest samples using the digestion type as a response, (**D**) PLS loading plot of LV1xLV2 featuring the 3349 most significant peptides from the linear model testing the response to proteases described in the Methods section, and coloured according to their retention time (min) and *m*/*z* values. T, trypsin/LysC, G, GluC, C, chymotrypsin, RT, retention time.

Statistical analyses were carried out on the volumes of the 27,123 peptides identified in this study. Multivariate analyses (PCA, PLS, HCA) were performed as well as a linear model which isolated 3349 peptides significantly responding to the digestion type (data not shown). The PCA projection plot of PC1 against PC2 using all identified peptides shows that samples are grouped by digestion type, with biological triplicate closely clustering together but technical duplicate separating out as they were run at two independent times (Figure 3A), which can be resolved by randomizing the LC injection order.

PC1 explains 35% of the total variance and separates samples that include digestion with trypsin/LysC on the right-hand side away from the samples which do not on the left-hand side. PC2 explains 11.3% of the variance and discriminates samples on the basis of their treatment with (below zero) or without (above zero) chymotrypsin (Figure 3A). Peptide mass is the determining factor behind the sample grouping across PC1xPC2 as can be seen on the PCA loading plot which illustrates that samples treated with GluC generate the longest peptides (>5 kDa, Figure 3B). A PLS analysis was performed using the 3349 peptides that were most significantly differentially expressed across the seven digestion types. This supervised statistical process helps better define groups according to a particular trait, in this instance the digestion type. The score plot of the first two components indeed achieves better separation of the different digestion types, with samples treated with GluC (G) away from all the other types (Figure 3C). One group is composed of the samples treated with trypsin/LysC (T) on its own and combined to GluC (T->G). Another group comprises samples treated with chymotrypsin on its own (C) and with GluC (G->C). The last group positioned in between contains samples treated with trypsin/LysC and chymotrypsin (T->C), as well as with GluC (T->G->C). The main peptide characteristics behind such grouping is the m/z value as illustrated on the PLS loading plot (Figure 3D). These types of multivariate analyses confirm the orthogonality of the proteases chosen in this experiment.

The number of MS peaks varies from 49,316 (Bud 2 T->G->C rep 2) to 118,020 (Bud 3 T->G rep 1), with an average value of 93,771 ± 15,426 (Table 2). 

The MS data of cannabis digests is less reproducible across technical replicates than across biological replicates due to the fact that technical replicates were run independently. With an average of 9 ± 11%, CVs range from 0.3% (Bud 3 C) to 33% (Bud 3 T) and achieve less than 7% in 15 out of the 21 digestion tests (Table 2). The number of MS/MS spectra obtained varied from 6,835 (Bud 2 T->G->C rep 1) to 12,827 (Bud 1 T rep 1), averaging 9072 ± 2047 which corresponds to 10% of the MS peaks. 

#### 2.2.2. Sequential Enzymatic Digestions of Medicinal Cannabis Samples Augment the Success Rate of MS/MS Annotations in SEQUEST

The MS data was searched against a *C. sativa* database using SEQUEST algorithm for protein identification purpose. Of all the MS/MS spectra generated from medicinal cannabis digests, between 824 (47% of the 1770 MS/MS spectra for Bud 2 T->G->C rep 2) and 4297 (38% of the 11,238 MS/MS spectra for Bud 3 T->C rep 1) are successfully annotated (Table 2). On average, 29% of the MS/MS spectra yield positive database hits, which amounts to an average of 2.7% of MS peaks. The list of peptides from medicinal cannabis identified in this study is available in Supplementary Table S2. 

The percentages of Table 2 are turned into a histogram in Supplementary Figure S6 to better visualize the proportions of useful data across proteases. As observed before for BSA samples, the proportion of MS peaks fragmented by MS/MS (light grey bars) remains fairly constant across the medicinal cannabis digests, ranging from 7–12% as it is set by the duty cycle. The proportion of MS/MS spectra annotated in SEQUEST (i.e., successful hits) however shows even more variation across proteases than reported on BSA above, fluctuating from 15% to 45% (black bars). Higher percentages are reached when chymotrypsin (C) is employed on its own or in combination with trypsin/LysC (T->C) and/or GluC (G->C and T->G->C) (Supplementary Figure S6). In the case of medicinal cannabis protein extracts, resorting to a strategy involving sequential enzymatic digestions using two or three proteases proves very successful with high annotation rates: 28% for T->G, 34% for G->C, 37% for T->C and 45% for T->G->C (Supplementary Figure S6). However, even with such a high success rate, at best, 5% of MS peaks lead to positive identifications meaning that 95% of the data remains untapped into. One strategy to deepen the proteome analysis in a similar data-dependent acquisition manner would be to re-analyse each sample several times using iterative exclusion lists of the precursor ions already fragmented. Another strategy would be to employ a data-independent acquisition strategy such as the sequential window acquisition of all theoretical mass spectra (SWATH-MS) [60].

**Figure 4:**
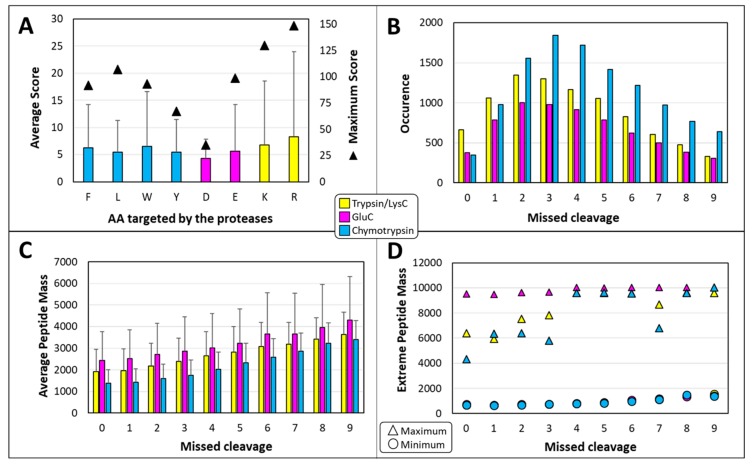
Statistics on medicinal cannabis peptides. (**A**) Averaged peptide ion score per AA residue targeted by the proteases. Maximum are represented by triangles. Vertical bars denote SDs. (**B**) Distribution of the numbers of missed cleavages per protease. (**C**) Distribution of the average masses of the cannabis peptides according to the number of missed cleavages. Vertical bars denote SDs. (**D**) Minimum (circles) and maximum (triangles) masses of the peptides according to the number of missed cleavages.

A total of 27,123 unique peptides from cannabis samples are identified in this study (Supplementary Table S2). This far exceeds what we had previously achieved using a BUP based on trypsin digestion where 5675 unique peptides across all replicates were successfully matched [11]. If we consider some the characteristics of the medicinal cannabis peptides identified in this study, we can see that each protease behaves differently. For instance, the highest peptide ion scores are found among the peptides generated by trypsin/LysC, in particular when R residues are targeted, whereas the lowest scores belong to peptides resulting from the cleavage of D residues upon the action of GluC. Ion scores average around 6.1 ± 9.6 and reach up to 148 (Figure 4A).

Out of the 27,123 cannabis peptides identified in this study, 80% (21,705) display one or multiple PTMs (Supplementary Table S2), including 4241 carbamidomethylation, 683 N-term acetylation, 18,716 phosphorylation and 9236 oxidation sites. Table 3 presents the distribution of PTMs per protease as imputed based on cleavage sites. 

Some annotated MS/MS spectra can be viewed in Figure S7. In these examples, peptides from ribulose bisphosphate carboxylase large chain (RBCL) are identified with high scores from GluC, chymotrypsin and trypsin/LysC (Figure S7A). MS/MS annotation from SEQUEST in Supplementary Figure S7B illustrates how each enzyme helps extend the coverage of RBCL spanning the region Y29 to R79 (YQTKDTDILAAFRVTPQPGVPPEEAGAAVAAESSTGTWTTVWTDGLTSLDR) with chymotrypsin covering residues 41–66, GluC extending the coverage to the left down to residue 29 and Trypsin/LysC extending it to the right up to residue 79. MS/MS spectra display almost complete b- and y-series ions (Figure S7B). RBCL is adorned with several dynamic PTMs, for instance oxidation of M116 (Figure S7C) and phosphorylation of T173 and Y185 (Figure S7D).

The distribution of identified cannabis peptides according to the number of missed cleavages also reveals differences among proteases. Our method specified a maximum of ten missed cleavage sites, which is the highest number allowed in the Proteome Discoverer program and SEQUEST algorithm. Missed cleavages have been discussed in the BSA results. All things considered, only 5% of the peptides present no missed cleavage and up to nine missed cleavages are detected in the MS/MS data (Figure 4B and Supplementary Table S2). The greatest numbers of peptides resulting from trypsin/LysC or GluC present two missed cleavages while the largest number of chymotryptic peptides possess three missed cleavages. Therefore, setting the correct parameters is essential to maximise the number of successful hits. Had we limited our search method to two missed cleavages as is traditionally performed in shotgun proteomics, 65% of the tryptic cannabis peptides would not have been identified. Furthermore, allowing for more missed cleavage has the intrinsic benefit of yielding longer peptides, befitting the middle-down range, which is very advantageous for sequencing purposes. Indeed, in the present work, average masses of cannabis peptides steadily increase with the number of enzymatic cleaving sites missed, in a similar manner for each of the proteases (Figure 4C). 

When we observe the minimum masses, we can see that they increase with the number of missed cleavages, very similarly across all three proteases (Figure 4D). The shortest cannabis peptide has a mass of 627.3956 Da (7 AAs, position 286–292, from Photosystem II protein D2), presents one miscleavage and arises from the action of chymotrypsin which is the least specific of the proteases tested in this experiment (Supplementary Table S2). When we observe the maximum masses, GluC systematically produce the largest peptides, fluctuating from 9479.692 to 10,027.014 Da, regardless of the number of missed cleavages (Figure 4D). The longest peptide has a mass of 10,0027.014 Da (88 AAs, position 57 to 144, from CBDA synthase), bears six missed cleavage sites and arises from the action of GluC which is the most specific of the proteases tested in this work (Supplementary Table S2). Trypsin/LysC and chymotrypsin display similar patterns, namely the maximum masses increase as the number of missed cleavages go from 0 to 4, and then plateau around 9.6 kDa for subsequent numbers of missed cleavages (Figure 4D). 

In our previous BUP study on medicinal cannabis, we set the maximum number of missed cleavages to two [11]; we have since re-analysed these BUP results by setting the number of miscleavages to ten (the maximum) and found 43 additional peptides containing 3–9 missed cleavages thus confirming valuable information was ignored (data not shown). We exemplify this in Supplementary Figure S8 which summarises the sequencing results for OAC, a key enzyme in the phytocannabinoid biosynthetic pathway. The gain in the number of OAC peptides identified here relative to our previous study [11] equals 31 additional peptides (PTMs included), many of them being longer therefore covering larger portions of the AA sequence (Figure S8A). Aligning the peptides along OAC sequence reveals that whilst complete coverage is achieved, more peptides from the N-terminus are identified relative to the C-terminus of the protein (Figure S8B). Trypsin/LysC yields 85% coverage of OAC, GluC 57% and chymotrypsin 53% (Figure S8C). In our previous BUP experiment only 34% coverage of OAC was reached using trypsin/LysC [11]; re-analysis with ten miscleavages brought OAC sequence coverage to 48% (data not shown). The complete 100% coverage of OAC observed here could only be achieved by combining the sequencing data associated with the four proteases together as none of them individually yield full coverage. This observation holds true for most proteins reported in this work.

#### 2.2.3. Proteins from Medicinal Cannabis Are Identified with High Sequence Coverage

The 27,123 cannabis peptides identified here are assigned to 494 unique accessions (Table S3) which corresponds to 229 unique proteins (Table S4) from *C. sativa* and close relatives. This comprises 130 (57%) novel protein annotations when the list is compared to that of our previous BUP study [11]. The molecular weight (MW) of these cannabis proteins average 38 ± 34 kDa, ranging from 2.8 kDa (Photosystem II phosphoprotein) to 271.2 kDa (Protein Ycf2). The AA sequence coverage varies from 6% (NAD(P)H-quinone oxidoreductase subunit J, chloroplastic) to 100% (108 out of 229 identities, 47%). The vast majority of the proteins (187/229, 82%) display a sequence coverage greater than 80% (Supplementary Table S4) which is a great improvement to those obtained previously [11]. Therefore, using different proteases on their own or in combination allowed the identification of more proteins with greater confidence. This has repeatedly been demonstrated on various complex biological samples [38,44,45,46,47,48,50,51,52,54,55]. Even though we did not prepare our samples with membrane proteins in mind (requiring specific membrane-disrupting chemicals such as sodium dodecyl sulphate), many of the identified accessions correspond to membrane proteins. They either display a transmembrane domain (141/494, 29%) and/or are localized to a biological membrane (100/494, 20%), mostly within chloroplasts (74%) (Supplementary Table S3). This Gene Ontology (GO) annotation is confirmed by elevated grand average of hydropathy (GRAVY) values (Supplementary Table S3). Chymotrypsin has been shown to help digest membrane proteins and therefore identify them by shotgun proteomics [61,62].

**Figure 5:**
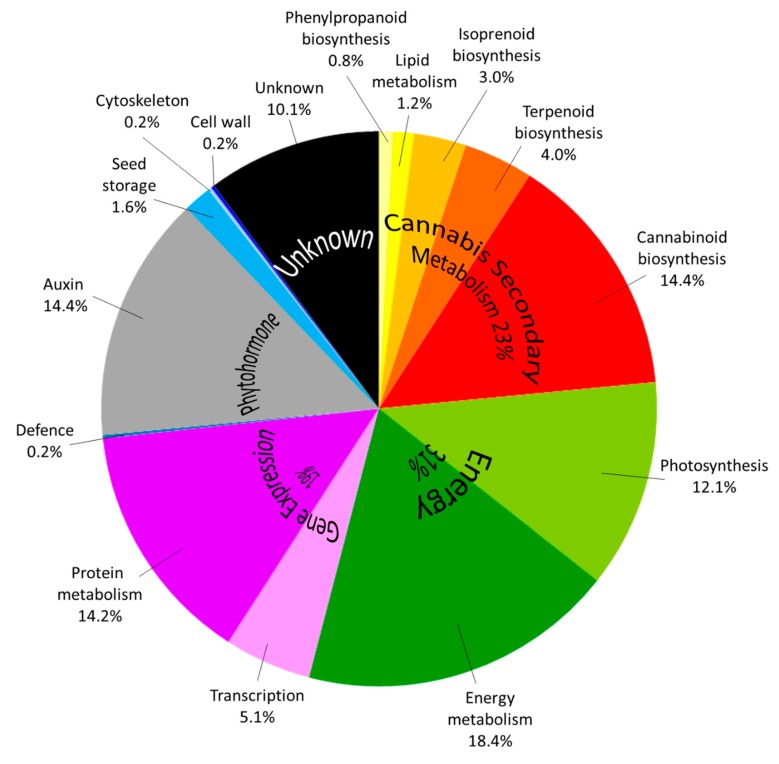
Pie chart of the pathways in which identified Cannabis proteins are involved.

As previously observed [11], the 494 cannabis protein accessions are predominantly involved in cannabis secondary metabolism (23%), energy production (31%, including photosynthesis 18%), and gene expression (19%, in particular protein metabolism 14%) (Figure 5). 

Using E.C. numbers, we performed a KEGG pathway mapping which highlights that all the phytocannabinoid-related enzymes and most of the enzymes involved in terpenoid backbone biosynthesis are identified in this study (Supplementary Figure S9). Ten percent of the proteins are of unknown function, including Cannabidiolic acid synthase-like 1 and 2 which display 84% similarity with CBDA synthase (data not shown). Most of the additional functions relative to [11] belong to the energy/photosynthesis pathway, translation mechanisms with many ribosomal proteins identified here (Supplementary Table S4), as well as a plethora (14.4%, 71 out of 494 accessions) of small auxin up regulated (SAUR) proteins (Supplementary Table S3). More significantly, all the enzymes involved in the cannabinoid biosynthetic pathway are identified and account for 14.4% of all the accessions (Figure 5) when they made only 5.6% of the accessions in [11]. Additional proteins from this pathway are three truncated products from THCA synthase of 11, 33, and 49 kDa, as well as polyketide synthases 1 to 5 whose AA sequences show 95% similarity to that of OLS (data not shown). Newly identified proteins include enzymes from the isoprenoid biosynthetic pathway: 2-C-methyl-d-erythritol 4-phosphate cytidylyltransferase and 3-hydroxy-3-methylglutaryl coenzyme A synthase. A naringenin-chalcone synthase involved in the biosynthesis of phenylpropanoids is also newly identified here. Finally, novel elements of the terpenoid pathway include (+)-alpha-pinene synthase and 2-acylphloroglucinol 4-prenyltransferase found in the chloroplast (Supplementary Table S4). This study demonstrates that combining different proteases is needed to achieve deeper recovery and a more thorough analysis of the proteins not only involved in the secondary metabolism of *C. sativa* but in the diverse biological mechanisms occurring in the mature buds of this unique species. With an ultimate goal of analyzing multiple medicinal cannabis samples, the method presented here utilizes a relatively fast (60 min) nLC elution to ensure high throughput. We anticipate that deeper proteome coverage would be reached with longer nLC gradient or pre-fractionation steps. 

*C. sativa* proteomics has come a long way since the first report in 2004 on reproductive organs and trichomes in which only 10 flower proteins and 54 gland proteins could be identified from *Arabidopsis* and rice accessions due to the absence of *C. sativa* database entries [19]. Ten years later in 2014, 481 proteins were identified from trichomes, with only 26 (5%) corresponding to *C. sativa* accessions [16]. The slow rate of entry creation from *C. sativa* species in public database was addressed in [11] where we highlighted that while the first *C. sativa* entry was created in 1986 in UniprotKB, by 2004 only six entries featured, and in 2014 entries amounted to 100. Most of *C. sativa* public entries (258) were uploaded in 2015–2017. There are currently (October 2019) 509 *C. sativa* protein accessions in UniprotKB. Early this year, we published a BUP study to optimize protein extraction from mature buds [11] in which 160 protein accessions were identified with 83% (133) of them matching a *C. sativa* accession. More recently, a 1-DE shotgun proteomics experiment showed that CBDA synthase and THCA synthases are secreted in trichome exudates and accumulated over the flowering period [20]. Using a TDP approach, we revealed previously unknown PTMs [12]. While progress has been made on cannabis proteomics, this area of research is still in its infancy and we hope that the pace will pick up in the future. Confirming the expression of *C. sativa* genes at the protein level will help validate the annotations of genome sequencing projects in a proteogenomic manner and facilitate breeding programs. 

## 3. Materials and Methods 

The experimental design is schematised in Figure 1.

### 3.1. Plant Materials and Chemicals

#### 3.1.1. Apical Bud Sampling and Grinding

Fresh plant material was obtained from the Victorian Government Medicinal Cannabis Cultivation Facility. Apical buds were excised using secateurs, snap frozen in liquid N_2_ and stored at –80 °C until grinding. Samples were collected from three different plants. Frozen buds were ground with a mortar and pestle in liquid N_2_. The powder was transferred into a 15 mL tube and stored at –80 °C until further use.

#### 3.1.2. Chemicals

All proteases were purchased from Promega (Alexandria, VIC, Australia): Trypsin/LysC mix (V5072, 100 µg), GluC (V1651, 50 µg), and Chymotrypsin (V106A, 25 µg). Albumin from bovine serum (BSA, A7906-10G, 98% pure) was purchased from Sigma-Aldrich Pty Ltd (North Ryde BC, NSW, Australia) and analysed by MS in previous studies [23,24]. Guanidine-HCl (98% purity) was purchased from Sigma-Aldrich Pty Ltd.

### 3.2. Protein Extraction Methods

The protein extraction was described in [11] and up-scaled for this experiment as the same sample would undergo various protease digestions. Briefly, 0.5 g of ground frozen powder was transferred into a 15 mL tube kept on ice pre-filled with 12 mL ice-cold 10% TCA/10mM DTT/acetone (*w*/*w*/*v*). Tubes were vortexed for 1 min and left at −20 °C overnight. The next day, tubes were centrifuged for 10 min at 5000 rpm and 4 °C. The supernatant was discarded, and the pellet was resuspended in 10 mL of ice-cold 10mM DTT/acetone (*w*/*v*) by vortexing for 1 min. Tubes were left at −20 °C for 2 h. The tubes were centrifuged as specified before and the supernatant discarded. This washing step of the pellets was repeated once more. The pellets were dried for 60 min under a fume hood. The dry pellets were resuspended in 2 mL of guanidine-HCl buffer (6M guanidine-HCl, 10 mM DTT, 5.37 mM sodium citrate tribasic dihydrate, and 0.1 M Bis-Tris) by vortexing for 1 min, sonicating for 10 min and vortexing for another minute. Tubes were incubated at 60 °C for 60 min. The tubes were centrifuged as described above and 1.8 mL of the supernatant was transferred into 2 mL microtubes. A 40 µL volume of 1M iodoacetamide (IAA)/water (*w*/*v*) solution was added to the tubes to achieve a final IAA concentration of 20 mM and to alkylate the DTT-reduced proteins. The tubes were vortexed for 1 min and left to incubate at room temperature in the dark for 60 min. 

A 10 mg/mL BSA solution was prepared in 1 mL of guanidine-HCl buffer. The tube was vortexed for 1 min and incubated at 60 °C for 60 min. A 20 µL volume of 1M IAA/water (*w*/*v*) solution was added to the tube to achieve a final IAA concentration of 20 mM. The BSA tube was vortexed for 1 min and left to incubate at room temperature in the dark for 60 min. 

### 3.3. Protein Assay

Protein extracts were diluted ten times using the guanidine-HCl buffer prior to the assay. The protein concentrations were measured in triplicates using the Pierce Microplate BCA protein assay kit (Thermo Fisher Scientific Australia Pty Ltd, Scoresby, VIC, Australia) following the manufacturer’s instructions. The BSA solution supplied in the kit (2 mg/mL) was used a standard. 

### 3.4. Protein Digestion

An aliquot corresponding to 100 μg of BSA or plant proteins was used for digestion with proteases. Digestions were performed following the manufacturer’s recommendations as detailed in the following subsections. We did not test different protease:protein ratios or different digestion buffers. We merely followed the manufacturer’s guidelines.

#### 3.4.1. Digestion Using a Trypsin/LysC Protease Mix (T)

The DTT-reduced and IAA-alkylated proteins were diluted six times using 50 mM Tris-HCl pH 8.0 to drop the guanidine-HCl resuspension buffer molarity to 1 M. Trypsin/LysC protease (Mass Spectrometry Grade, 100 μg, Promega, Alexandria, VIC, Australia) was carefully solubilised in 1 mL of 50 mM acetic acid and incubated at 37 °C for 15 min. A 40 µL aliquot of trypsin/LysC solution was added and gently mixed with the protein extracts thus achieving a 1:25 ratio of protease mix:proteins, as instructed by the manufacturer. The mixture was left to incubate overnight (18 h) at 37 °C in the dark. 

#### 3.4.2. Digestion Using GluC (G)

The DTT-reduced and IAA-alkylated proteins were diluted six times using 50 mM Ammonium bicarbonate (pH 7.8) to drop the guanidine-HCl resuspension buffer molarity to 1 M. Under these conditions, GluC protease (Mass Spectrometry Grade, 50 μg, Promega) presents a greater specificity towards E residues. GluC was carefully solubilised in 0.5 mL of ddH_2_O. A 10 µL aliquot of GluC solution was added and gently mixed with the protein extracts thus achieving a 1:100 ratio of protease:proteins. The mixture was left to incubate overnight (18 h) at 37 °C in the dark. 

#### 3.4.3. Digestion Using Chymotrypsin (C)

The DTT-reduced and IAA-alkylated proteins were diluted six times using 100 mM Tris/10mM CaCl_2_ pH 8.0 to drop the guanidine-HCl resuspension buffer molarity to 1 M. Chymotrypsin protease (Sequencing Grade, 25 μg, Promega) was carefully solubilised in 0.25 mL of 1 M HCl. A 10 µL aliquot of chymotrypsin solution was added and gently mixed with the protein extracts, thus achieving a 1:100 ratio of protease:proteins. The mixture was left to incubate overnight (18 h) at 25 °C in the dark. 

#### 3.4.4. Sequential Digestions Using Several Proteases (G->C, T->G, T->C, T->G->C)

Digestion using GluC was performed as described above. A 10 µL aliquot of Chymotrypsin solution was then added, gently mixed with the GluC digest, and incubated at 25 °C in the dark for 18h. This yielded the digest we refer to as G->C. Digestion using Trypsin/LysC was performed as described above. A 10 µL aliquot of GluC or Chymotrypsin solution was then added and gently mixed with the trypsin/LysC digest. The tubes were incubated in the dark for 18 h at 37 °C for GluC or 25 °C for Chymotrypsin. These steps yielded the digests we refer to as T->G or T->C. For the sequential digestion combining all proteases, to the T->G sample was added a 10 µL aliquot of Chymotrypsin solution and incubated at 25 °C in the dark for 18h. This yielded the digest we refer to as T->G->C.

#### 3.4.5. Equimolar Mixtures of Digests (T:G, T:G, G:C, T:G:C)

In an effort to assess the efficiency of the sequential digestions (T->G, T->G, G->C, T->G->C), individual BSA digests resulting from the independent activity of Trypsin/LysC, GluC and Chymotrypsin were pooled together using the same volumes. Thus, the Trypsin/LysC digest was pooled with the GluC digest (T:G), the Trypsin/LysC digest was pooled with the Chymotrypsin digest (T:C), the GluC digest was pooled with the Chymotrypsin digest (G:C), and the three Trypsin/Lys, GluC and Chymotrypsin digests were also pooled together (T:G:C).

### 3.5. Desalting

All of the digestion reactions were stopped by lowering the pH of the mixture using a 10% formic acid (FA) in H_2_O (*v*/*v*) to a final concentration of 1% FA.

All digests were desalted using solid phase extraction (SPE) cartridges (Sep-Pak C18 1cc Vac Cartridge, 50 mg sorbent, 55–105 μm particle size, 1 mL, Waters, Rydalmere, NSW, Australia) by gravity, followed by Speedvac evaporation as described in [24].

The digest was transferred into a 100 μL glass insert placed into a glass vial. The vials were positioned into the autosampler at 4 °C for immediate analyses by nLC-MS/MS.

### 3.6. Peptide Digest Analysis by Nano Liquid Chromatography-Tandem Mass Spectrometry (nLC-MS/MS)

The nLC-ESI-MS/MS analyses were performed on all the peptide digests in duplicate. Chromatographic separation of the peptides was performed by reverse phase (RP) using an Ultimate 3000 RSLCnano System (Dionex, Thermo Fisher Scientific Australia Pty Ltd) online with an Elite Orbitrap hybrid ion trap-Orbitrap mass spectrometer (Thermo Fisher Scientific Australia Pty Ltd). The parameters for nLC and MS/MS have been described in [24] and [11]. Briefly, 0.1 μg peptides were separated by nLC with a 3% to 40% B gradient over 35 min. Full MS scans were acquired over a mass range of 300 to 2000 *m*/*z* with a 60,000 resolution in profile mode. The 20 most intense peaks with charge state ≥ 2 and a minimum signal threshold of 10,000 were MS/MS fragmented in the linear ion trap using collision-induced dissociation (CID). Dynamic exclusion was enabled, and peaks selected for fragmentation more than once within 10 s were excluded from selection for 30 s. Each digest was injected twice, with first injecting all the digests (technical replicate 1) and then fully repeating the injections in the same order (technical replicate 2). 

### 3.7. Database Search for Protein Identification and Annotation

This proof-of-concept work aims at demonstrating the gain in sequence coverage, hence protein identities, yielded upon the use of multiple proteases relative to our previous study [11]. To this end, we employ the same database search strategy. However, more sequences can be retrieved from genome sequencing projects [8,9,10], the National Center for Biotechnology Information (NCBI) website, and the Medicinal Plant Genomic Resource (MPGR) website. This will be achieved in our future experiments. Database searching of the RAW files was performed in Proteome Discoverer (PD) 1.4 using SEQUEST algorithm as described in [11]. All 668 *Cannabis sativa* protein sequences publicly available in October 2019 from UniprotKB (www.uniprot.org; key word used “Cannabis sativa”, https://www.uniprot.org/uniprot/?query=taxonomy:3744%20cannabis%20sativa) were downloaded as a FASTA file. These also included 87 sequences from the European hop *Humulus lupulus*, the closest relative to *C. sativa* [63], as well as 72 sequences from the Chinese grass *Boehmeria nivea* also closely related to cannabis [63]. Because the GOT sequence was not included, we retrieved it from patent WO 2011/017798 Al [64] and included it in the FASTA file (669 entries, available as Supplementary Data). The FASTA file was imported and indexed in PD 1.4. The SEQUEST algorithm was used to search the indexed FASTA file. The database searching parameters specified trypsin, or GluC, or chymotrypsin or their respective combinations as the digestion enzymes and allowed for up to ten missed cleavages. The precursor mass tolerance was set at 10 ppm, and fragment mass tolerance set at 0.8 Da. Peptide absolute Xcorr threshold was set at 0.4, the fragment ion cutoff was set at 0.1%, and protein relevance threshold was set at 1.5. Carbamidomethylation (C) was set as a static modification and oxidation (M), phosphorylation (STY), and N-Terminus acetylation were set as dynamic modifications. The target decoy peptide-spectrum match (PSM) validator was used to estimate false discovery rates (FDR). At the peptide level, peptide confidence value set at high was used to filter the peptide identification, and the corresponding FDR on peptide level was less than 1%. At the protein level, protein grouping was enabled. 

All nLC-MS/MS files are available from the stable public repository MassIVE at the following URL: http://massive.ucsd.edu/ProteoSAFe/datasets.jsp with the accession number MSV000084216. Using the FASTA sequences of the identified accessions, GRAVY values were retrieved online from https://www.bioinformatics.org/sms2/protein_gravy.html. Likewise, GO subcellular localizations were retrieved online from the UniprotKB Retrieve/ID mapping webpage (https://www.uniprot.org/uploadlists/). Enzyme E.C. numbers were retrieved from UniprotKB and BRENDA websites (https://www.brenda-enzymes.org/). Pathway analysis was performed by uploading enzyme E.C. numbers into the online pathway mapping tool of KEGG website (https://www.genome.jp/kegg/tool/color_a_pathway.html).

### 3.8. Data Processing and Statistical Analyses

#### 3.8.1. nLC-MS/MS Data Processing

The data files obtained following nLC-MS/MS analysis were processed in the Refiner MS module of Genedata Expressionist^®^ 12.0 with the following parameters: (1) Load from file by restricting the range from 8–45 min, (2) metadata import, (3) Spectrum smoothing using moving average algorithm and a minimum of 5 points, (4) RT structure removal using a minimum of 3 scans, (5) *m*/*z* grid using an adaptative grid method with a scan count of 10 and a 10% smoothing, (6) chromatogram RT alignment with a pairwise alignment based tree, a maximum shift of 50 scans and no gap penalty, (7) chromatogram peak detection using a 10 scan summation window, a 0.1 min minimum peak size, 0.04 Da maximum merge distance, a boundaries merge strategy, a 20% gap/peak ratio, a curvature-based algorithm, intensity-weighed and using inflection points to determine boundaries, (8) MS/MS consolidation, (9) Proteome Discoverer import accepting only top-ranked database matches and no decoy results, (10) peak annotation, (11) export analyst using peak volumes and implicit logarithm (treating zeros as missing values).

A peptide mapping activity for BSA digest samples was also performed using the mature AA sequence of the protein (P02769|25-607) following step 8 (MS/MS consolidation) as follows: (12) Selection of the relevant protease digests, (13) peptide mapping using the following parameters: 10 ppm mass tolerance, ESI-CID/HCD instrument, 0.8 Da fragment tolerance, min fragment score of 30, top-ranked only, discard mass-only matches, enzymes varied according to the protease(s) used, 6 max missed cleavages, min peptide length of 3, fixed carbamidomethyl (C) modification, and variable oxidation (M) modification.

#### 3.8.2. Statistical Analyses

Statistical analyses were performed using the Analyst module of Genedata Expressionist^®^ 12.0 where columns denote plant samples and rows denote digest peptides. Peak volumes exported from the Refiner module were used as a proxy of peptide quantities for all statistical analyses. Principal Component Analyses (PCA) were performed on rows using a covariance matrix with 40% valid log-transformed values and row mean as imputation. A linear model performed on rows and testing the digestion type. Partial Least Square (PLS) analyses were run on the most significant rows resulting from the linear model. PLS response was the digestion type with three latent factors, 50% valid values and row mean as imputation. Hierarchical clustering analysis (HCA) was performed on columns using positive correlation and Ward linkage method. Histograms were generated by exporting the number of peaks, number of MS/MS spectra, and masses of the identified peptides to a Microsoft excel 2016 (office 365) spreadsheet.

## Figures and Tables

**Table 1:** Number of MS peaks, MS/MS spectra and MS/MS spectra annotated with SEQUEST for each of the bovine serum albumin (BSA) digests. For protease legend, refer to Figure 1. An arrow (->) indicates the order in which the proteases were added. A colon (:) indicates that individual digests were pooled with equimolarity.

Tube		1. MS					2. all MS/MS				% MS/MS	3. SEQUEST Annotated MS/MS				% MS/MS Annotated	% MS Annotated
Sample	Protease Mix	Rep 1	Rep 2	Mean	SD	% CV	Rep 1	Rep 2	Mean	SD	Percent	Rep 1	Rep 2	Mean	SD	%	%
BSA	T	83,678	83,056	83,367	440	0.5	9769	9325	9547	314	11	2133	1875	2004	182	21	2.4
BSA	G	91,922	98,895	95,409	3487	3.7	9081	9628	9355	387	10	929	1363	1146	307	12	1.2
BSA	C	92,116	90,303	91,210	907	1.0	10,327	9792	10,060	378	11	1358	1267	1313	64	13	1.4
BSA	T->G	89,648	83,107	86,378	3271	3.8	11,311	9698	10,505	1141	12	2178	1978	2078	141	20	2.4
BSA	T:G	84,347	87,462	85,905	1558	1.8	8605	9720	9163	788	11	2141	2332	2237	135	24	2.6
BSA	T->C	87,203	79,616	83,410	3794	4.5	10,944	8810	9877	1509	12	1864	1549	1707	223	17	2.0
BSA	T:C	90,847	92,736	91,792	945	1.0	10,245	10,115	10,180	92	11	2428	1931	2180	351	21	2.4
BSA	G->C	77,085	82,055	79,570	2485	3.1	6450	5163	5807	910	7	1103	475	789	444	14	1.0
BSA	G:C	99,001	100,001	99,501	500	0.5	9980	9847	9914	94	10	1169	1065	1117	74	11	1.1
BSA	T->G->C	88,919	84,798	86,859	2061	2.4	9880	6137	8009	2647	9	1485	1005	1245	339	16	1.4
BSA	T:G:C	91,975	89,420	90,698	1278	1.4	10,201	9503	9852	494	11	1015	1616	1316	425	13	1.5
BSA	mean	88,795	88,314	88,554	1884	2	9708	8885	9297	796	10	1618	1496	1557	244	17	2
BSA	SD	5707	6752	5811	1218	1	1317	1648	1333	756	1	544	531	501	136	4	1
	min	77,085	79,616	79,570	440	1	6450	5163	5807	92	7	929	475	789	64	11	1
	max	99,001	100,001	99,501	3794	5	11,311	10,115	10,505	2647	12	2428	2332	2237	444	24	3

**Table 2:** Number of MS peaks, MS/MS spectra and MS/MS spectra annotated in SEQUEST for each of the medicinal cannabis digests. For protease legend, refer to Figure 1. An arrow (->) indicates the order in which the proteases were added.

Tube		1. MS					2. all MS/MS				% MS/MS	3. SEQUEST Annotated MS/MS					% MS/MS Annotated	% MS Annotated
Biol rep	Protease mix	Rep 1	Rep 2	Mean	SD	% CV	Rep 1	Rep 2	Mean	SD	Percent	Rep 1	Rep 2		Mean	SD	%	%
Bud 1	T	86,458	115,577	101,018	20,590	20.4	12,827	11,731	12,279	775	12	2042	1929	1986		80	16	2.0
Bud 2	T	72,907	113,303	93,105	28,564	30.7	10,775	11,160	10,968	272	12	1606	1740	1673		95	15	1.8
Bud 3	T	70,473	112,818	91,646	29,942	32.7	10,541	10,585	10,563	31	12	1513	1643	1578		92	15	1.7
Bud 1	G	106,622	84,761	95,692	15,458	16.2	9035	8501	8768	378	9	1388	1376	1382		8	16	1.4
Bud 2	G	95,761	88,387	92,074	5214	5.7	8032	7906	7969	89	9	1200	1146	1173		38	15	1.3
Bud 3	G	93,760	91,846	92,803	1353	1.5	8810	8115	8463	491	9	1326	1290	1308		25	15	1.4
Bud 1	C	93,117	95,399	94,258	1614	1.7	9486	8644	9065	595	10	2589	2200	2395		275	26	2.5
Bud 2	C	93,778	92,536	93,157	878	0.9	8433	7788	8111	456	9	2232	1857	2045		265	25	2.2
Bud 3	C	97,359	97,813	97,586	321	0.3	9508	8341	8925	825	9	2382	2098	2240		201	25	2.3
Bud 1	T->G	116,131	113,352	114,742	1965	1.7	11,909	11,406	11,658	356	10	3416	3163	3290		179	28	2.9
Bud 2	T->G	113,690	111,601	112,646	1477	1.3	11,511	10,857	11,184	462	10	3103	2904	3004		141	27	2.7
Bud 3	T->G	118,020	115,958	116,989	1458	1.2	12,362	11,811	12,087	390	10	3633	3405	3519		161	29	3.0
Bud 1	T->C	98,125	94,395	96,260	2638	2.7	10,963	9568	10,266	986	11	4066	3434	3750		447	37	3.9
Bud 2	T->C	98,455	97,615	98,035	594	0.6	10,622	9090	9856	1083	10	4024	3308	3666		506	37	3.7
Bud 3	T->C	100,667	97,679	99,173	2113	2.1	11,238	8873	10,056	1672	10	4297	3321	3809		690	38	3.8
Bud 1	G->C	92,277	90,930	91,604	952	1.0	8219	7625	7922	420	9	2786	2545	2666		170	34	2.9
Bud 2	G->C	86,056	83,949	85,003	1490	1.8	7160	6390	6775	544	8	2393	2190	2292		144	34	2.7
Bud 3	G->C	93,847	89,624	91,736	2986	3.3	8158	7398	7778	537	8	2687	2502	2595		131	33	2.8
Bud 1	T->G->C	88,886	56,861	72,874	22,645	31.1	9479	4279	6879	3677	9	4117	2002	3060		1496	44	4.2
Bud 2	T->G->C	67,123	49,316	58,220	12,591	21.6	6835	1770	4303	3581	7	3065	824	1945		1585	45	3.3
Bud 3	T->G->C	84,077	77,062	80,570	4960	6.2	7685	5570	6628	1496	8	3392	2524	2958		614	45	3.7
	Mean	13,559	17,773	13,095	9797	11	1743	2526	2047	992	1	991	787	836		439	10	1
	SD	13,232	17,345	12,779	9561	11	1701	2465	1997	968	1	967	769	816		428	10	1
	Min	67,123	49,316	58,220	321	0.33	6835	1770	4303	31.1	7.391	1200	824	1173		8.49	14.7195	1.27398
	Max	118,020	115,958	116,989	29,942	32.7	12,827	11,811	12,279	3677	12.155	4297	3434	3809		1585	45.1894	4.19837

**Table 3:** Number of fixed and dynamic PTMs per protease.

Proteases	Carbamidomethylation	Acetylation	Phosphorylation	Oxidation	Total
Trypsin/LysC	1362	296	6213	2927	10,798
Chymotrypsin	1483	238	7683	3520	12,924
GluC	1396	149	4820	2789	9154
Total	4241	683	18,716	9236	32,876

## Supplementary Materials

Supplementary materials can be found at https://www.mdpi.com/1422-0067/20/22/5630/s1. Supplementary Figure S1: LC-MS patterns of BSA digests. Supplementary Figure S2: MS peaks statistics from BSA samples. Supplementary Figure S3: AA composition of BSA. Supplementary Figure S4: Distribution of BSA peptide masses according to the number of miscleavages per digestion combinations. Supplementary Figure S5: LC-MS patterns of digests from medicinal cannabis buds. Supplementary Figure S6: MS peaks statistics from medicinal cannabis samples. Supplementary Figure S7: SEQUEST annotation of MS/MS spectra of some peptides from ribulose bisphosphate carboxylase large chain (RBCL, UniprotID A0A0C5B2I6). Supplementary Figure S8: Sequencing results for olivetolic acid cyclase (OAC, uniprotID I6WU39, 101 AA residues). Supplementary Figure S9: Cannabis enzymes involved in terpenoid (A) and cannabinoid (B) metabolisms. Supplementary Table S1: List of BSA peptides resulting from various proteases and identified by shotgun proteomics. Supplementary Table S2: List of medicinal cannabis peptides resulting from various proteases and identified by shotgun proteomics. Supplementary Table S3: List of medicinal cannabis accessions identified by shotgun proteomics. Supplementary Table S4: List of medicinal cannabis unique proteins identified by shotgun proteomics.

## Abbreviations

AA	amino acid
ACN	acetonitrile
BSA	bovine serum albumin
BUP	bottom-up proteomics
CBCA	cannabichromenic acid
CBDA	cannabidiolic acid
CBGA	cannabigerolic acid
FDR	false discovery rate
GluC	glutamyl peptidase I
GOT	geranylpyrophosphate:olivetolate geranyltransferase
HCA	hierarchical clustering analysis
kDa	kiloDalton
LysC	lysyl endopeptidase
MDP	middle-down proteomics
MS	mass spectrometry
MS/MS	tandem mass spectrometry
MW	molecular weight
nLC	nano-liquid chromatography
OAC	olivetolic acid cyclase
OLS	3,5,7-trioxododecanoyl-CoA synthase
PCA	principal component analysis
PLS	partial least square
PTM	post-translational modification
RBCL	ribulose bisphosphate carboxylase large chain
SD	standard deviation
TDP	top-down proteomics
THCA	delta9-tetrahydrocannabinolicacid

## References

[1] Andre C.M., Hausman J.-F., Guerriero G. (2016). *Cannabis sativa*: The plant of the thousand and one molecules. Front. Plant Sci..

[2] ElSohly M.A., Slade D. (2005). Chemical constituents of marijuana: The complex mixture of natural cannabinoids. Life Sci..

[3] Brenneisen R. (2007). Chemistry and Analysis of Phytocannabinoids and Other Cannabis Constituents.

[4] Fischedick J.T., Hazekamp A., Erkelens T., Choi Y.H., Verpoorte R. (2010). Metabolic fingerprinting of *Cannabis sativa* l., cannabinoids and terpenoids for chemotaxonomic and drug standardization purposes. Phytochemistry.

[5] Radwan M.M., Elsohly M.A., Slade D., Ahmed S.A., Khan I.A., Ross S.A. (2009). Biologically active cannabinoids from high-potency *Cannabis sativa*. J. Nat. Prod..

[6] Adams R., Hunt M., Clark J.H. (1940). Structure of cannabidiol, a product isolated from the marihuana extract of minnesota wild hemp. J. Am. Chem. Soc..

[7] Mechoulam R., Gaoni Y. (1967). Recent advances in the chemistry of hashish. Prog. Chem. Org. Nat. Prod..

[8] Grassa C.J., Wenger J.P., Dabney C., Poplawski S.G., Motley S.T., Michael T.P., Schwartz C.J., Weiblen G.D. (2018). A complete cannabis chromosome assembly and adaptive admixture for elevated cannabidiol (CBD) content. bioRxiv.

[9] Laverty K.U., Stout J.M., Sullivan M.J., Shah H., Gill N., Holbrook L., Deikus G., Sebra R., Hughes T.R., Page J.E. (2018). A physical and genetic map of *Cannabis sativa* identifies extensive rearrangement at the thc/cbd acid synthase locus. Genome Res..

[10] van Bakel H., Stout J.M., Cote A.G., Tallon C.M., Sharpe A.G., Hughes T.R., Page J.E. (2011). The draft genome and transcriptome of *Cannabis sativa*. Genome Biol..

[11] Vincent D., Rochfort S., Spangenberg G. (2019). Optimisation of protein extraction from medicinal cannabis mature buds for bottom-up proteomics. Molecules.

[12] Vincent D., Binos S., Rochfort S., Spangenberg G. (2019). Top-down proteomics of medicinal cannabis. Proteomes.

[13] Aiello G., Fasoli E., Boschin G., Lammi C., Zanoni C., Citterio A., Arnoldi A. (2016). Proteomic characterization of hempseed (*Cannabis sativa* L.). J. Proteom..

[14] Behr M., Sergeant K., Leclercq C.C., Planchon S., Guignard C., Lenouvel A., Renaut J., Hausman J.F., Lutts S., Guerriero G. (2018). Insights into the molecular regulation of monolignol-derived product biosynthesis in the growing hemp hypocotyl. BMC Plant Biol..

[15] Bona E., Marsano F., Cavaletto M., Berta G. (2007). Proteomic characterization of copper stress response in *Cannabis sativa* roots. Proteomics.

[16] Happyana N. (2014). Metabolomics, Proteomics, and Transcriptomics of Cannabis sativa L. Trichomes. Ph.D. Thesis.

[17] Mamone G., Picariello G., Ramondo A., Nicolai M.A., Ferranti P. (2019). Production, digestibility and allergenicity of hemp (*Cannabis sativa* L.) protein isolates. Food Res. Int..

[18] Park S.K., Seo J.B., Lee M.Y. (2012). Proteomic profiling of hempseed proteins from cheungsam. Biochim. Biophys. Acta.

[19] Raharjo T.J., Widjaja I., Roytrakul S., Verpoorte R. (2004). Comparative proteomics of *Cannabis sativa* plant tissues. J. Biomol. Tech..

[20] Rodziewicz P., Loroch S., Marczak L., Sickmann A., Kayser O. (2019). Cannabinoid synthases and osmoprotective metabolites accumulate in the exudates of *Cannabis sativa* L. Glandular trichomes. Plant Sci..

[21] Xia C., Hong L., Yang Y., Yanping X., Xing H., Gang D. (2019). Protein changes in response to lead stress of lead-tolerant and lead-sensitive industrial hemp using swath technology. Genes.

[22] Raynes J.K., Vincent D., Zawadzki J.L., Savin K., Mertens D., Logan A., Williams R.P.W. (2018). Investigation of age gelation in uht milk. Beverages.

[23] Vincent D., Elkins A., Condina M.R., Ezernieks V., Rochfort S. (2016). Quantitation and identification of intact major milk proteins for high-throughput lc-esi-q-tof ms analyses. PLoS ONE.

[24] Vincent D., Ezernieks V., Elkins A., Nguyen N., Moate P.J., Cocks B.G., Rochfort S. (2015). Milk bottom-up proteomics: Method optimization. Front. Genet..

[25] Vincent D., Mertens D., Rochfort S. (2018). Optimisation of milk protein top-down sequencing using in-source collision-induced dissociation in the maxis quadrupole time-of-flight mass spectrometer. Molecules.

[26] Meyer B., Papasotiriou D.G., Karas M. (2011). 100% protein sequence coverage: A modern form of surrealism in proteomics. Amino Acids.

[27] Switzar L., Giera M., Niessen W.M.A. (2013). Protein digestion: An overview of the available techniques and recents developments. J. Proteome Res..

[28] Tsiatsiani L., Heck A.J. (2015). Proteomics beyond trypsin. FEBS J..

[29] Vandermarliere E., Mueller M., Martens L. (2013). Getting intimate with trypsin, the leading protease in proteomics. Mass Spectrom. Rev..

[30] Olsen J.V., Ong S.E., Mann M. (2004). Trypsin cleaves exclusively c-terminal to arginine and lysine residues. Mol. Cell Proteom..

[31] Gershon P.D. (2014). Cleaved and missed sites for trypsin, lys-c, and lys-n can be predicted with high confidence on the basis of sequence context. J. Proteome Res..

[32] Slechtova T., Gilar M., Kalikova K., Tesarova E. (2015). Insight into trypsin miscleavage: Comparison of kinetic constants of problematic peptide sequences. Anal. Chem..

[33] Glatter T., Ludwig C., Ahrne E., Aebersold R., Heck A.J., Schmidt A. (2012). Large-scale quantitative assessment of different in-solution protein digestion protocols reveals superior cleavage efficiency of tandem lys-c/trypsin proteolysis over trypsin digestion. J. Proteome Res..

[34] Klammer A.A., MacCoss M.J. (2006). Effects of modified digestion schemes on the identification of proteins from complex mixtures. J. Proteome Res..

[35] Wisniewski J.R. (2016). Quantitative evaluation of filter aided sample preparation (FASP) and multienzyme digestion fasp protocols. Anal. Chem..

[36] Wisniewski J.R., Mann M. (2012). Consecutive proteolytic digestion in an enzyme reactor increases depth of proteomic and phosphoproteomic analysis. Anal. Chem..

[37] Drapeau G.R., Boily Y., Houmard J. (1972). Purification and properties of an extracellular protease of staphylococcus aureus. J. Biol. Chem..

[38] Cristobal A., Marino F., Post H., van den Toorn H.W., Mohammed S., Heck A.J. (2017). Toward an optimized workflow for middle-down proteomics. Anal. Chem..

[39] Nesvizhskii A.I., Aebersold R. (2005). Interpretation of shotgun proteomic data: The protein inference problem. Mol. Cell Proteom..

[40] Moradian A., Kalli A., Sweredoski M.J., Hess S. (2014). The top-down, middle-down, and bottom-up mass spectrometry approaches for characterization of histone variants and their post-translational modifications. Proteomics.

[41] Sidoli S., Garcia B.A. (2017). Middle-down proteomics: A still unexploited resource for chromatin biology. Expert Rev. Proteom..

[42] Holt M.V., Wang T., Young N.L. (2019). One-pot quantitative top- and middle-down analysis of gluc-digested histone h4. J. Am. Soc. Mass Spectrom..

[43] Belov A.M., Zang L., Sebastiano R., Santos M.R., Bush D.R., Karger B.L., Ivanov A.R. (2018). Complementary middle-down and intact monoclonal antibody proteoform characterization by capillary zone electrophoresis - mass spectrometry. Electrophoresis.

[44] MacCoss M.J., McDonald W.H., Saraf A., Sadygov R., Clark J.M., Tasto J.J., Gould K.L., Wolters D., Washburn M., Weiss A. (2002). Shotgun identification of protein modifications from protein complexes and lens tissue. Proc. Natl. Acad. Sci. USA.

[45] Schlosser A., Vanselow J.T., Kramer A. (2005). Mapping of phosphorylation sites by a multi-protease approach with specific phosphopeptide enrichment and nanolc-ms/ms analysis. Anal. Chem..

[46] Trevisan-Silva D., Bednaski A.V., Fischer J.S.G., Veiga S.S., Bandeira N., Guthals A., Marchini F.K., Leprevost F.V., Barbosa V.C., Senff-Ribeiro A. (2017). A multi-protease, multi-dissociation, bottom-up-to-top-down proteomic view of the loxosceles intermedia venom. Sci. Data.

[47] Fischer F., Poetsch A. (2006). Protein cleavage strategies for an improved analysis of the membrane proteome. Proteome Sci..

[48] Fischer F., Wolters D., Rogner M., Poetsch A. (2006). Toward the complete membrane proteome: High coverage of integral membrane proteins through transmembrane peptide detection. Mol. Cell Proteom..

[49] Zhang X. (2015). Less is more: Membrane protein digestion beyond urea-trypsin solution for next-level proteomics. Mol. Cell Proteom..

[50] Biringer R.G., Amato H., Harrington M.G., Fonteh A.N., Riggins J.N., Hühmer A.F. (2006). Enhanced sequence coverage of proteins in human cerebrospinal fluid using multiple enzymatic digestion and linear ion trap lc-ms/ms. Brief Funct. Genom. Proteom..

[51] Choudhary G., Wu S.L., Shieh P., Hancock W.S. (2003). Multiple enzymatic digestion for enhanced sequence coverage of proteins in complex proteomic mixtures using capillary lc with ion trap ms/ms. J. Proteome Res..

[52] Nagaraj N., Wisniewski J.R., Geiger T., Cox J., Kircher M., Kelso J., Paabo S., Mann M. (2011). Deep proteome and transcriptome mapping of a human cancer cell line. Mol. Syst. Biol..

[53] Giansanti P., Tsiatsiani L., Low T.Y., Heck A.J. (2016). Six alternative proteases for mass spectrometry-based proteomics beyond trypsin. Nat. Protoc..

[54] Guo X., Trudgian D.C., Lemoff A., Yadavalli S., Mirzaei H. (2014). Confetti: A multiprotease map of the hela proteome for comprehensive proteomics. Mol. Cell Proteom..

[55] Swaney D.L., Wenger C.D., Coon J.J. (2010). Value of using multiple proteases for large-scale mass spectrometry-based proteomics. J. Proteome Res..

[56] Gouveia D.D., Silva A.M., Vitorino R., Domingues M.R., Domingues P. (2014). The efficiency of trypsin digestion for mass-spectrometry-based identification and quantification of oxidized proteins: Evaluation of the digestion of oxidized bovine serum albumin. Eur. J. Mass Spectrom..

[57] Liang L.H., Liu C.C., Chen B., Yan L., Yu H.L., Yang Y., Wu J.N., Li X.S., Liu S.L. (2019). Lc-hrms screening and identification of novel peptide markers of ricin based on multiple protease digestion strategies. Toxins.

[58] Cottrell J.S. (2011). Protein identification using ms/ms data. J. Proteom..

[59] Stead D.A., Preece A., Brown A.J. (2006). Universal metrics for quality assessment of protein identifications by mass spectrometry. Mol. Cell Proteom..

[60] Gillet L.C., Navarro P., Tate S., Rost H., Selevsek N., Reiter L., Bonner R., Aebersold R. (2012). Targeted data extraction of the ms/ms spectra generated by data-independent acquisition: A new concept for consistent and accurate proteome analysis. Mol. Cell Proteom..

[61] Giannone R.J., Wurch L.L., Podar M., Hettich R.L. (2015). Rescuing those left behind: Recovering and characterizing underdigested membrane and hydrophobic proteins to enhance proteome measurement depth. Anal. Chem..

[62] Vermachova M., Purkrtova Z., Santrucek J., Jolivet P., Chardot T., Kodicek M. (2011). New protein isoforms identified within arabidopsis thaliana seed oil bodies combining chymotrypsin/trypsin digestion and peptide fragmentation analysis. Proteomics.

[63] McPartland J.M. (2018). Cannabis systematics at the levels of family, genus, and species. Cannabis Cannabinoid. Res..

[64] Page J., Boubakir Z. (2011). Aromatic Prenyltransferase from Cannabis.

