# Supplementary material for: A Multiple Protease Strategy to Optimise the Shotgun Proteomics of Mature Medicinal Cannabis Buds
Source: Int J Mol Sci. 2019 Nov 11;20(22):5630. doi: 10.3390/ijms20225630 (PMC6888629; doi:10.3390/ijms20225630)

Vincent D, Ezernieks V, Rochfort S, and Spangenberg G

"A multiple protease strategy to optimise shotgun proteomics of medicinal cannabis mature buds"

Supplementary Figures

Supplementary Figure S1: LC-MS patterns of BSA digests. T, single digestion using trypsin/lysC mix; G, single digestion using GluC; C, single digestion using chymotrypsin; T->G, double sequential digestion using first trypsin/lysC then GluC; T->C, double sequential digestion using first trypsin/lysC then chymotrypsin; double G->C, sequential digestion using first GluC then chymotrypsin; T->G->C, triple sequential digestion using first trypsin/lysC then GluC and finally chymotrypsin; T:G, equimolar mixture of trypsin/lysC and GluC single digests; T:C, equimolar mixture of trypsin/lysC and chymotrypsin single digests; G:C, equimolar mixture of GluC and chymotrypsin single digests; T:G:C, equimolar mixture of trypsin/lysC ,GluC and chymotrypsin single digests; X-axis 300-1600 m/z; y-axis 9-39 min; LC-MS peak intensity is indicated by a colour scale on LC-MS maps from purple, blue, yellow and red, the latter denoting the highest signal. The histogram bars represent the number of MS peaks detected on each LC-MS file averaged across technical replicates of digestions. Vertical lines denote standard deviations. Black diamonds represent the averaged number of MS/MS spectra per digestion. These data are also available on Table 1.

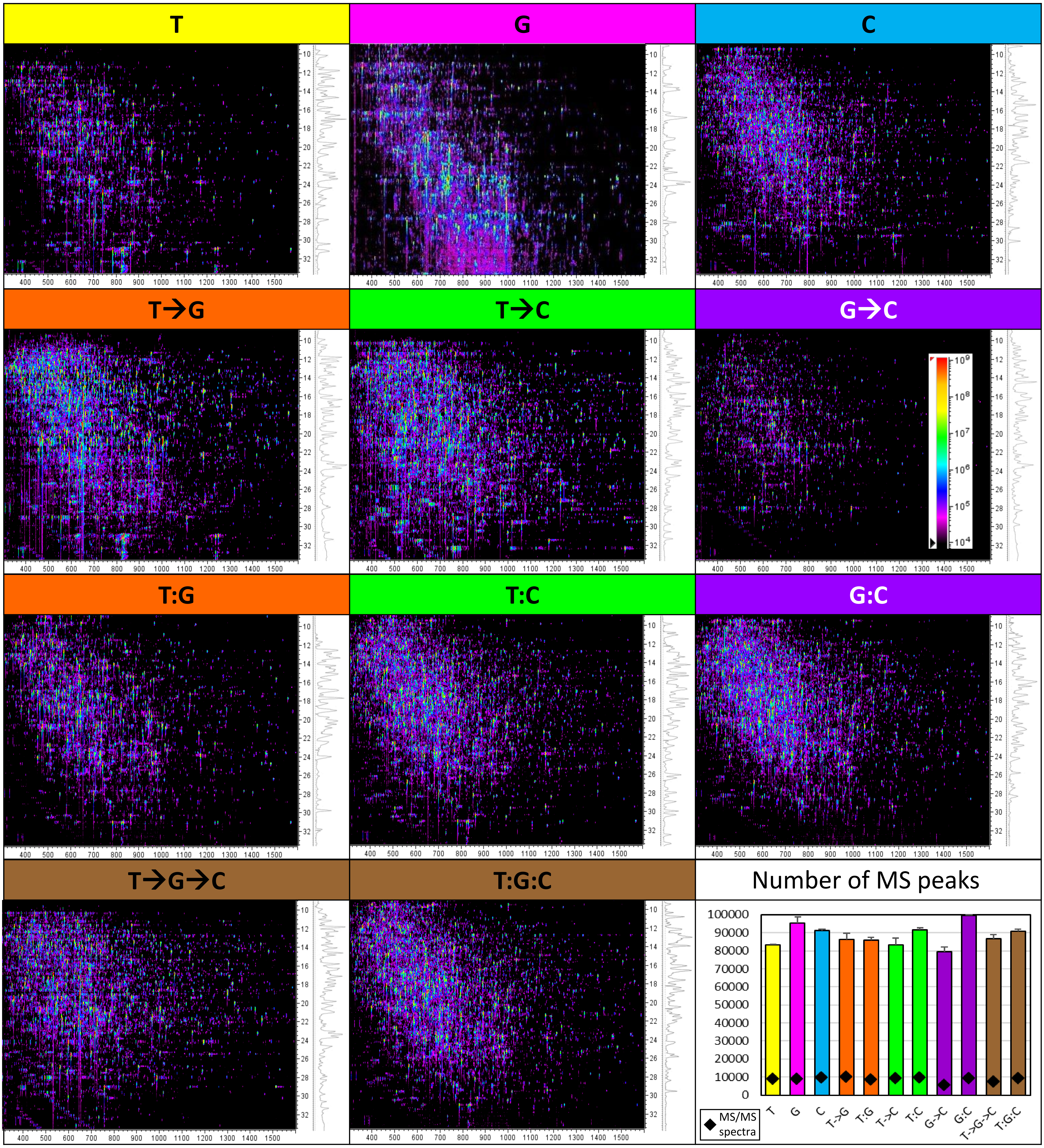

Supplementary Figure S2: MS peaks statistics from BSA samples. Percentages of MS peaks that underwent MS/MS fragmentation, of MS/MS spectra that were annotated using SEQUEST algorithm, and of MS peaks that led to an identification in SEQUEST. Values sourced from Table 1.

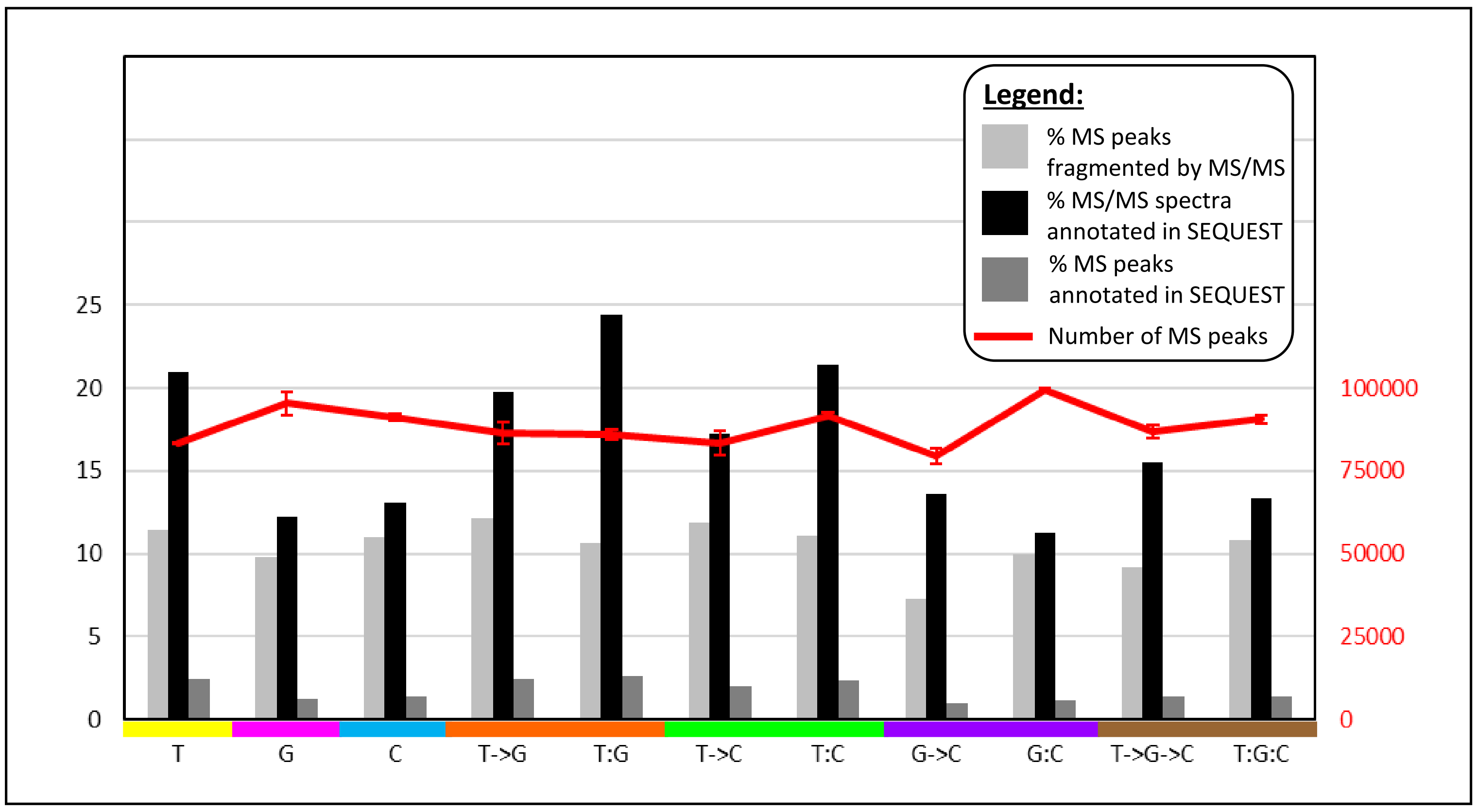

Supplementary Figure S3: AA composition of BSA. A. theoretical AA composition obtained using BSA mature protein sequence into Expasy ProtParam program (https://web.expasy.org/cgi-bin/protparam/protparam); B. comparison of predicted and observed cleavage sites.

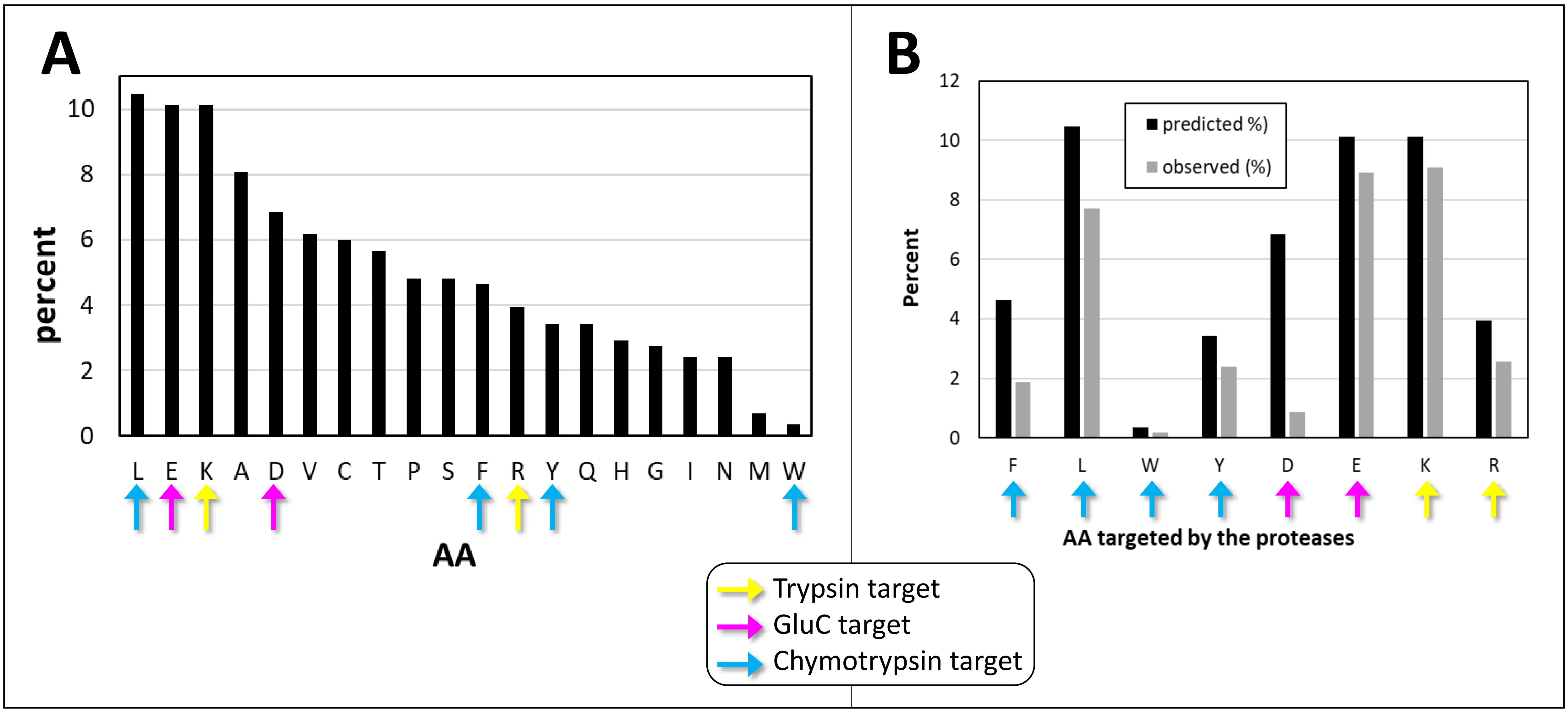

Supplementary Figure S4: Distribution of percentage of BSA peptide according to the number of miscleavages per digestion combinations.

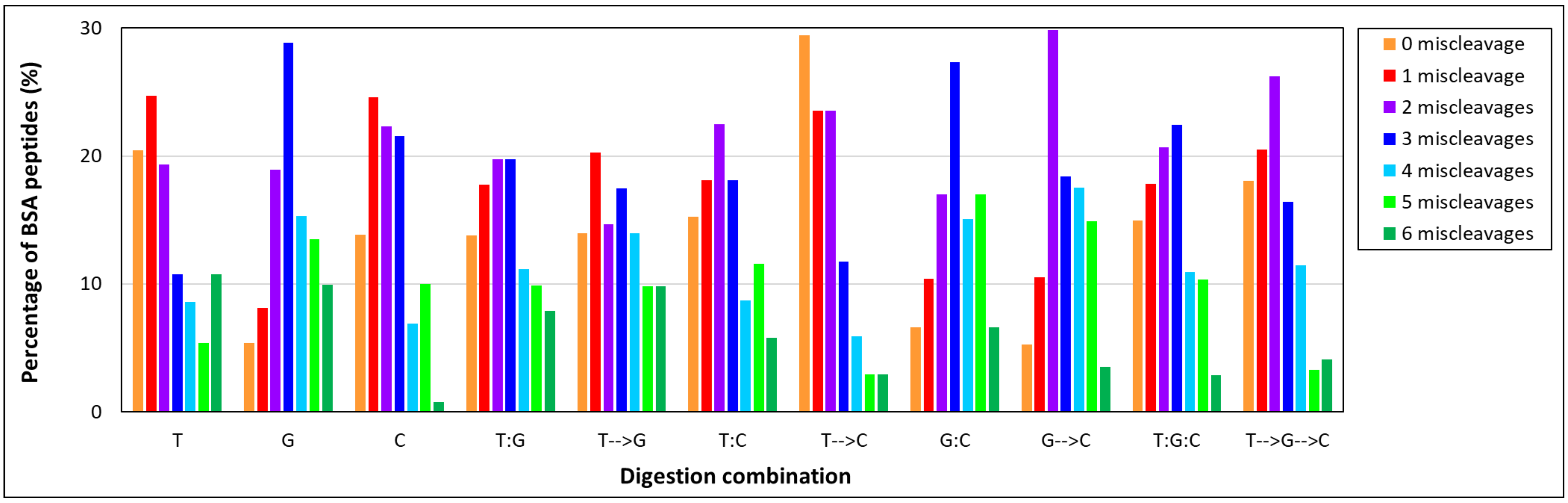

Supplementary Figure S5: LC-MS patterns of digests from medicinal cannabis buds. T, single digestion using trypsin/lysC mix; G, single digestion using GluC; C, single digestion using chymotrypsin; T->G, double sequential digestion using first trypsin/lysC then GluC; T->C, double sequential digestion using first trypsin/lysC then chymotrypsin; double G->C, sequential digestion using first GluC then chymotrypsin; T->G->C, triple sequential digestion using first trypsin/lysC then GluC and finally chymotrypsin; T:G, equimolar mixture of trypsin/lysC and GluC single digests; T:C, equimolar mixture of trypsin/lysC and chymotrypsin single digests; G:C, equimolar mixture of GluC and chymotrypsin single digests; T:G:C, equimolar mixture of trypsin/lysC ,GluC and chymotrypsin single digests, the horizontal black areas correspond to nanospray interruption; X-axis 300-1700 m/z; y-axis 9-39 min; LC-MS peak intensity is indicated by a colour scale on LC-MS maps from purple, blue, yellow and red, the latter denoting the highest signal. The histogram bars represent the number of MS peaks detected on each LC-MS file averaged across technical replicates. Vertical lines denote standard deviations. Black diamonds represent the averaged number of MS/MS spectra across technical replicates. These data are also available on Table 2.

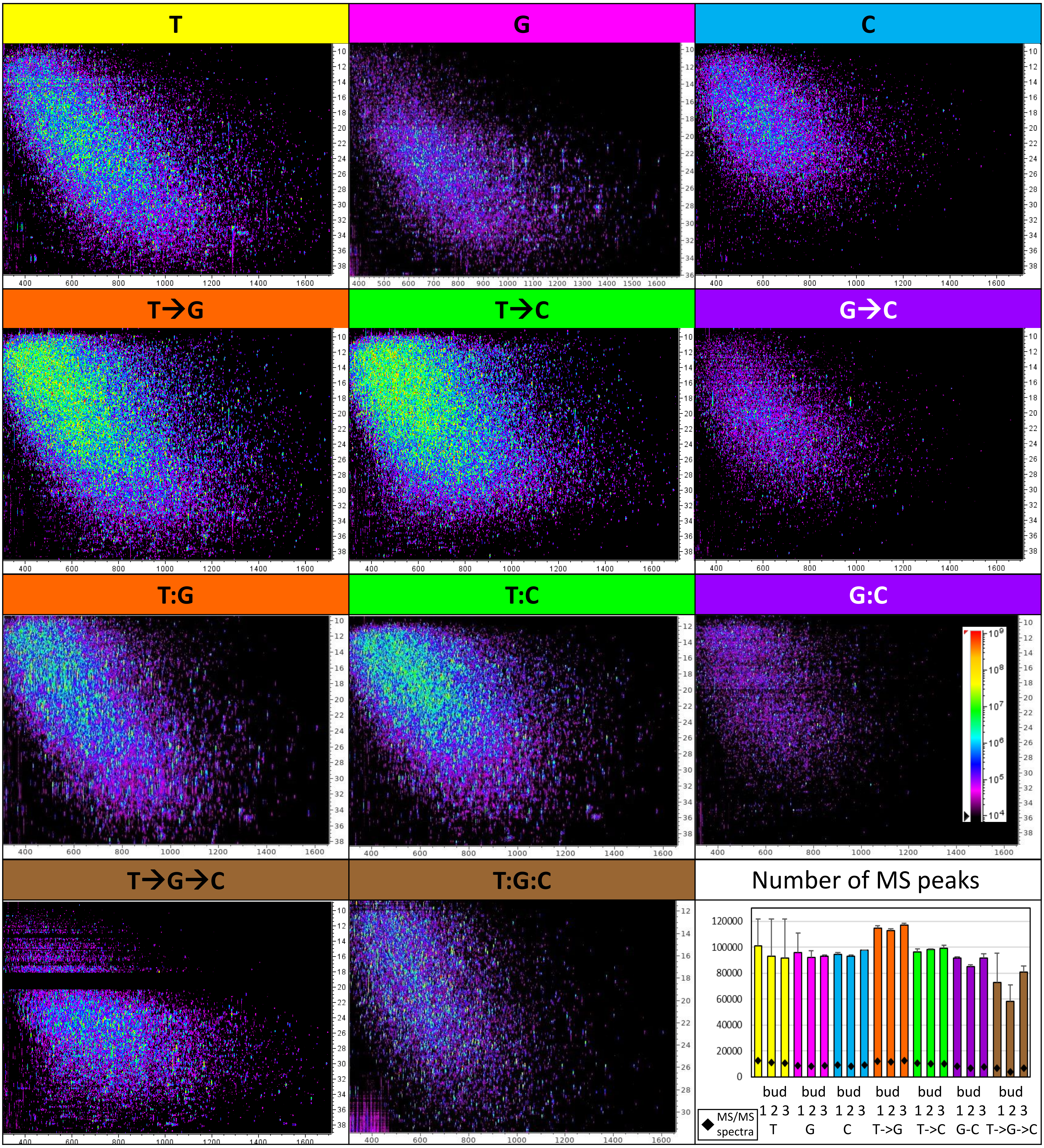

Supplementary Figure S6: MS peak statistics from medicinal cannabis samples. Percentages of MS peaks that underwent MS/MS fragmentation, of MS/MS spectra that were annotated using SEQUEST algorithm, and of MS peaks that led to an identification in SEQUEST. Values sourced from Table 2.

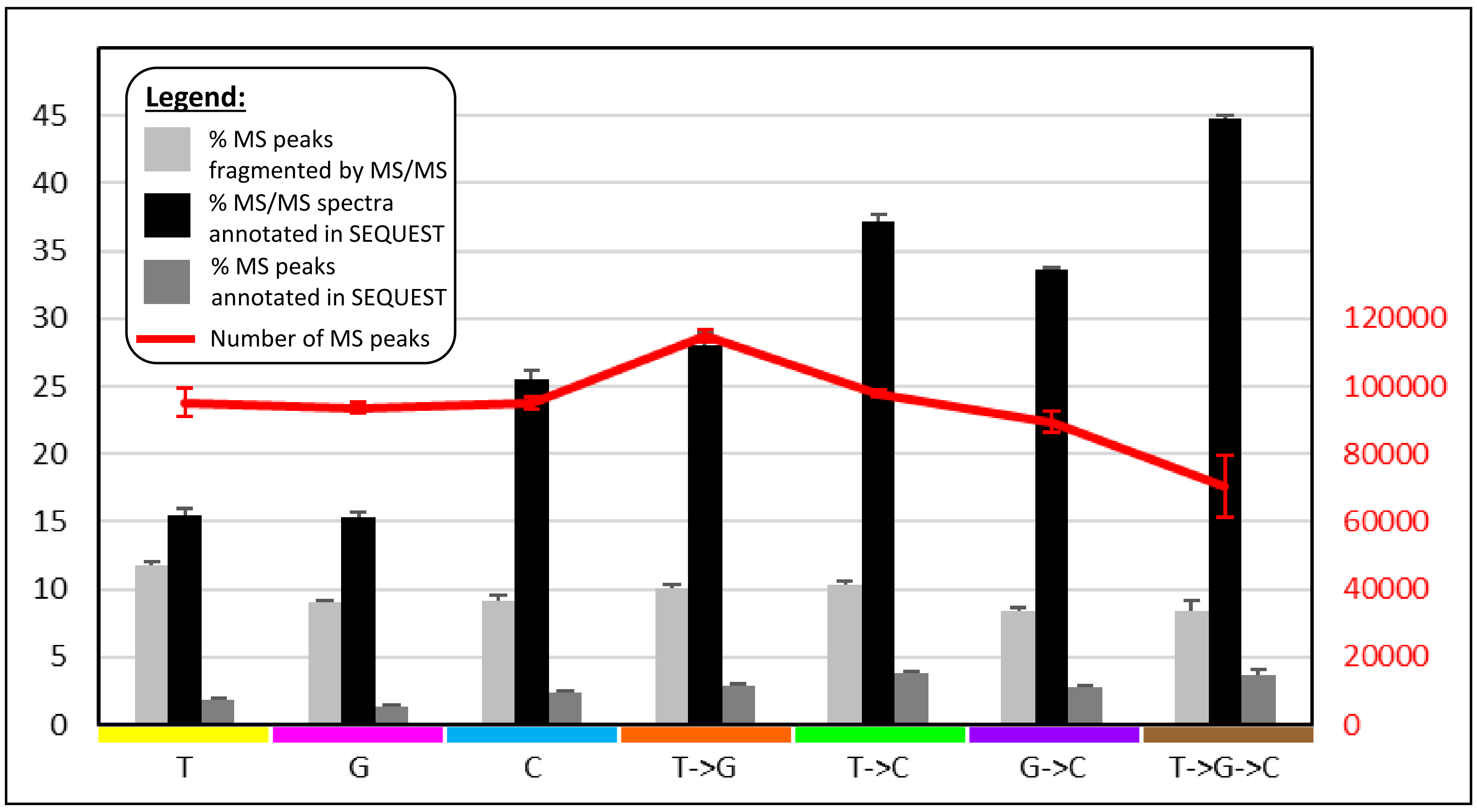

Supplementary Figure S7: SEQUEST annotation of MS/MS spectra of some peptides from ribulose bisphosphate carboxylase large chain (RBCL, UniprotID A0A0C5B2I6). A. Features of the peptides chosen to illustrate MS/MS annotation. B. Comparison of the same RBCL sequence area resulting from the action of trypsin/LysC, GluC or chymotrypsin. C-D. Examples of PTM annotations such as oxidation (C) or phosphorylations (D).

A

Peak	Spectrum	Peptide	Modifications	Protease	Start pos	End pos	Length	m/z	Calc. Mass	Charge	Mass Delta	Ion Score	Identity Score	Missed Cleavage	Ion Matches	Ion Count
Peak_159772	Spectrum_5594	YQTKDTDILAAFRVTPQPGVPPEEAGAAVAAE		GluC	29	60	32	1105.23	3311.67	3	-0.0025	51.79	16.90	4	19	169
Peak_176341	Spectrum_4795	RVTPQPGVPPEEAGAAVAAESSTGTW		Chymotrypsin	41	66	26	1283.64	2564.25	2	0.0095	70.35	20.61	0	22	85
Peak_176531	Spectrum_08454	VTPQPGVPPEEAGAAVAAESSTGTWTTVWTDGLTSLDR		Trypsin/LysC	42	79	38	1286.30	3853.86	3	0.0229	49.77	18.69	0	31	136
Peak_135431	Spectrum_09773	GSVTNMFTSIVGNVFGFK		Trypsin/LysC	111	128	18	953.49	1903.95	2	0.0074	108.41	18.92	0	11	15
Peak_136958	Spectrum_08435	GSVTNMFTSIVGNVFGFK	Oxidation [M6]	Trypsin/LysC	111	128	18	961.49	1919.95	2	0.0082	89.26	20.61	0	14	93
Peak_000562	Spectrum_0016	LGCTIKPK	Carbamidomethyl [C3]	Trypsin/LysC	170	177	8	306.18	915.52	3	-0.0026	21.51	19.19	1	8	20
Peak_038178	Spectrum_1006	LGCTIKPKLGLSAKNYGR	Carbamidomethyl [C3] Phospho [T4 Y16]	Trypsin/LysC	170	187	18	534.77	2135.04	4	-0.0105	15.37	26.14	3	4	10

B

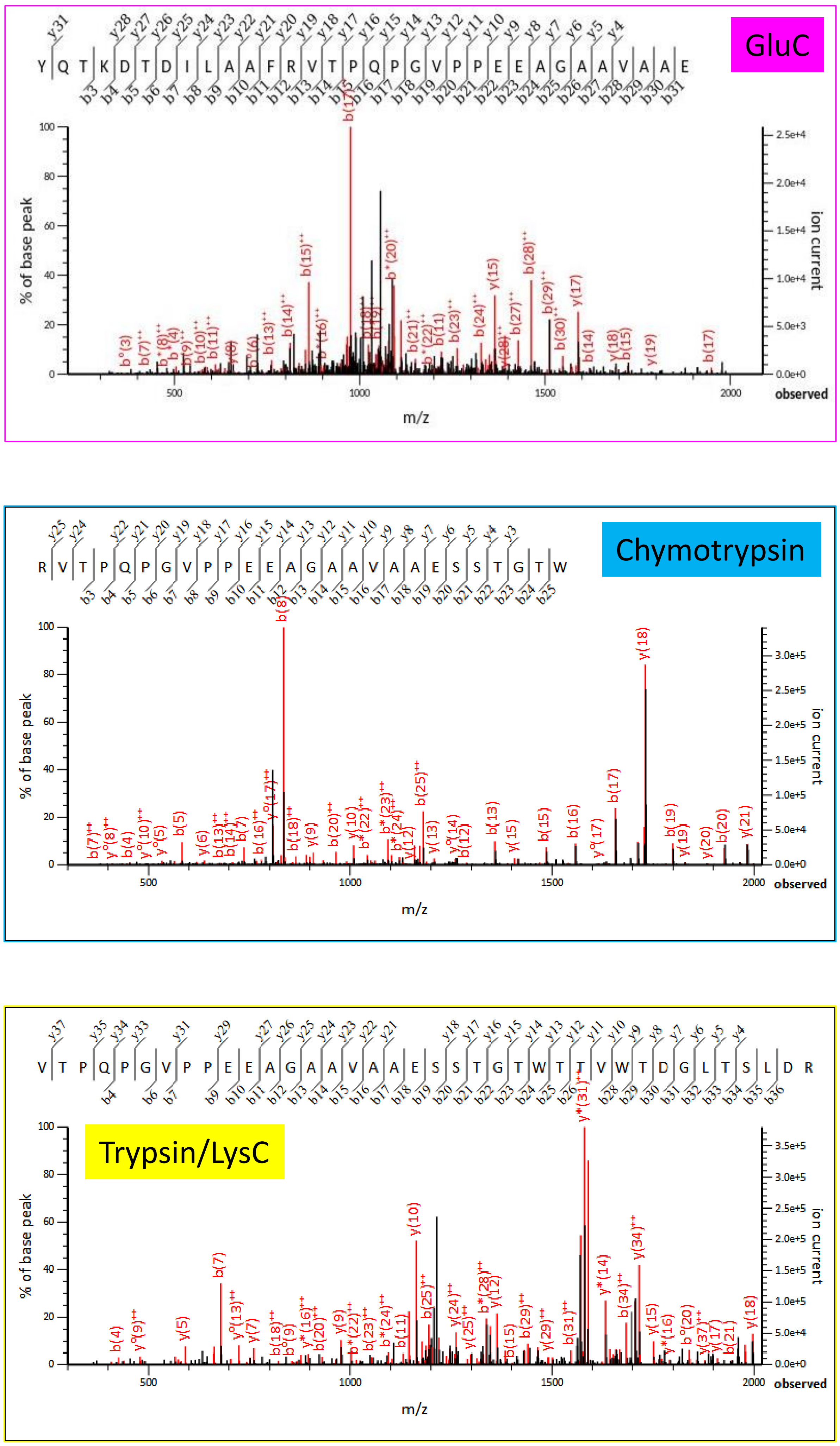

Peptide alignment:

YQTKDTDILAAFRVTPQPGVPPEEAGAAVAAE-----------------------------------

-------------------RVTPQPGVPPEEAGAAVAAESSTGTW-----------------------

---------------------VTPQPGVPPEEAGAAVAAESSTGTWTTVWTDGLTSLDR

C

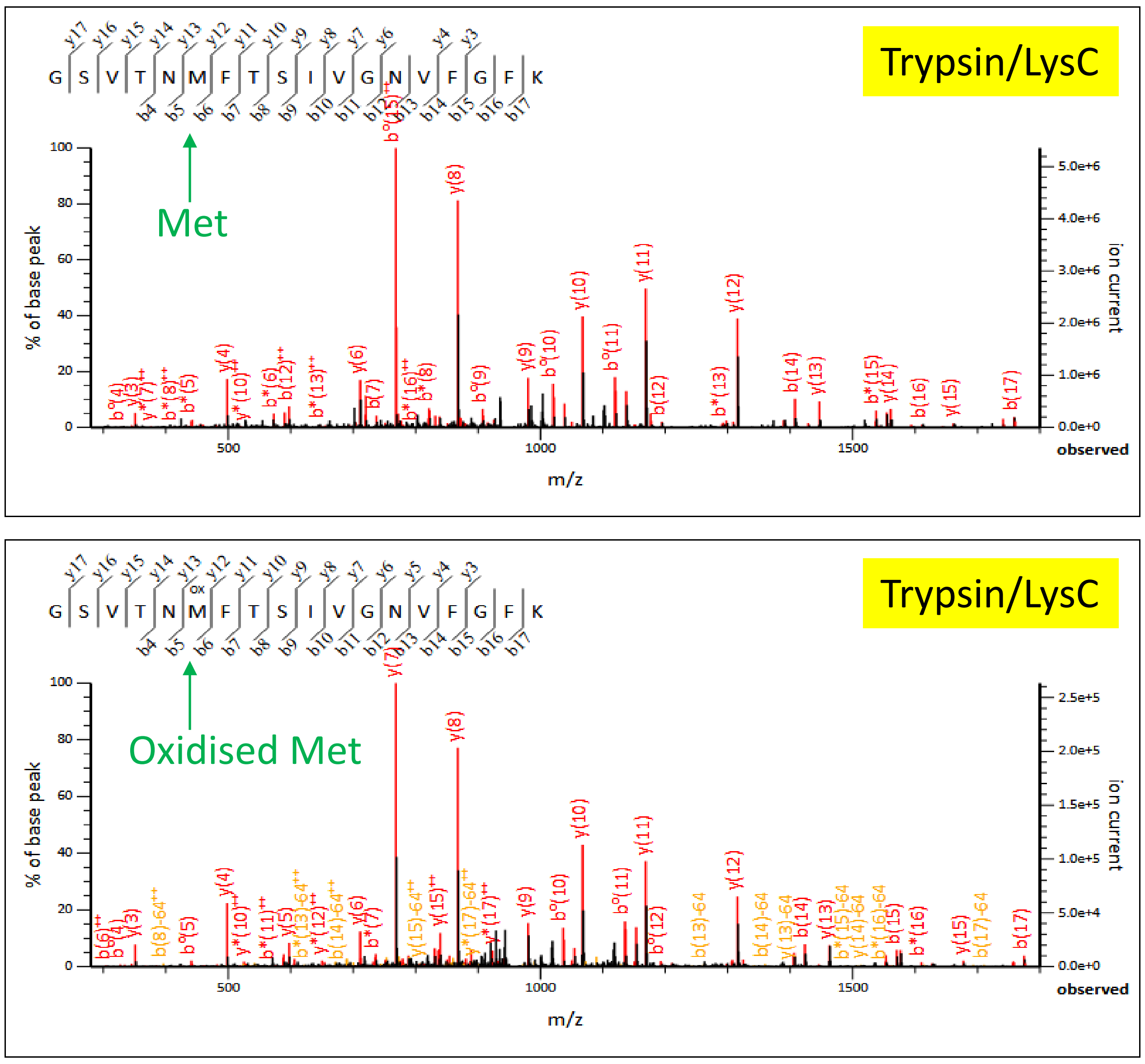

D

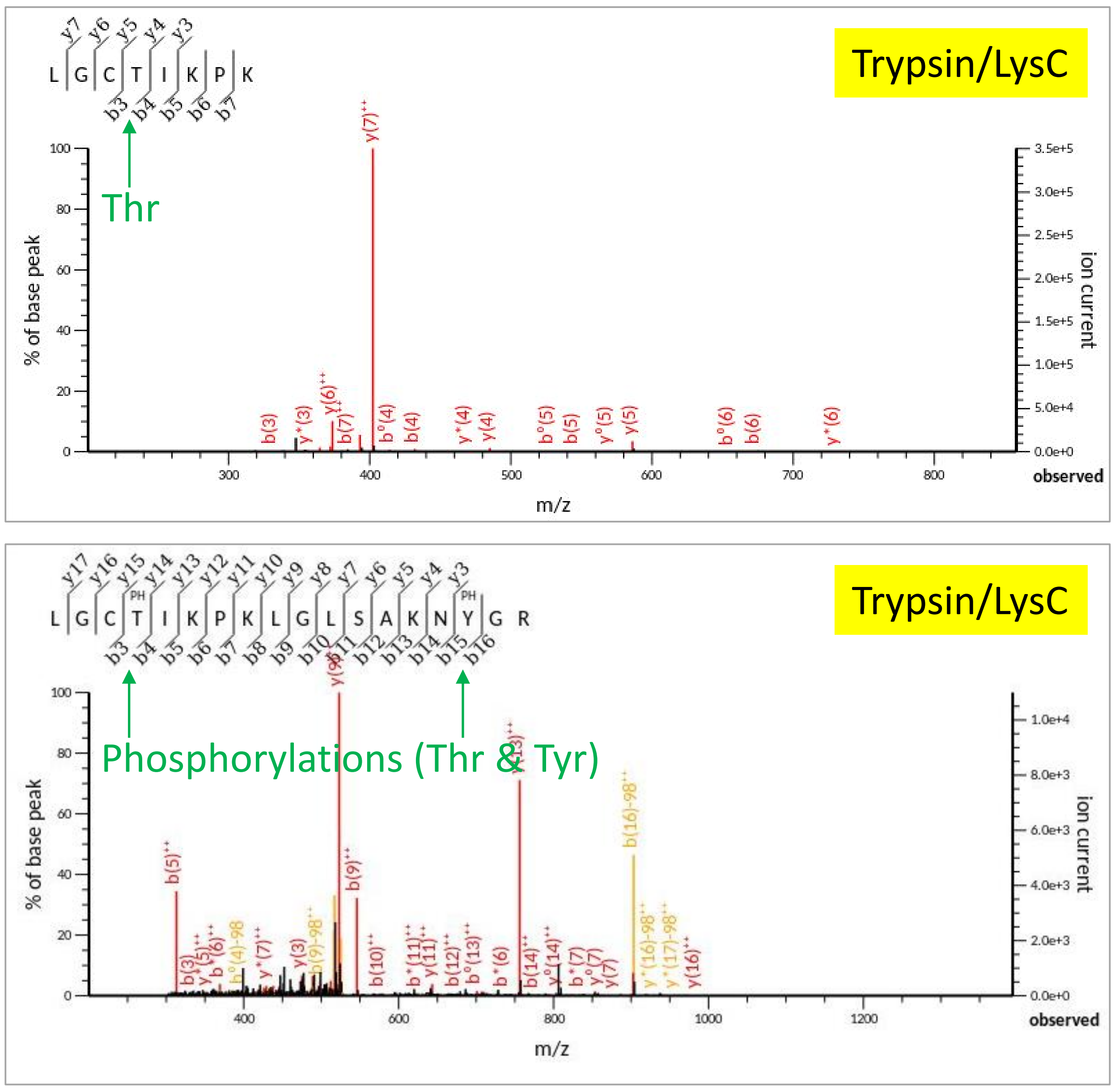

Supplementary Figure S8: **Sequencing results for olivetolic acid cyclase (OAC, uniprotID I6WU39, 101 AA residues)**. A. Features of the peptides identified in this study and in Vincent *et al*. 2019 [1]; B. Bioedit alignment of the identified peptides along the whole AA sequence of OAC; C. Sequencing summary for each study.

A

Study	Unique Peptide	Modifications	Charge	m/z	RT	Mass	Start Pos	End Pos	Peptide length	Missed Cleavages	Score	Protease
This study	MAVKHLIVLKFKDEITE	Oxidation [M1]	3	677.36	37.17	2029.13	1	17	17	8	4.27	GluC
This study	AVKHLIVLKFKDE		4	385.72	16.64	1538.92	2	14	13	1	29.37	GluC
This study	HLIVLKFKDEITEAQKE	Phospho [T12]	4	531.60	13.82	2120.09	5	21	17	6	2.23	GluC
This study	KFKDEITEAQKEEF		3	581.31	17.26	1740.86	10	23	14	1	22.29	Chymotrypsin
This study	FKDEITEAQKEEF	Phospho [T6]	4	424.21	34.08	1692.73	11	23	13	3	2.11	Chymotrypsin
This study	FKDEITEAQKEEFFKTYVNLVNIIPAMK	Oxidation [M27] Phospho [T6]	4	861.18	44.00	3440.70	11	38	28	3	1.14	Trypsin\LysC
This study	DEITEAQKEEFFKTYVNLVNIIPAMK	Phospho [T4 Y15]	4	808.35	22.80	3229.51	13	38	26	7	7.24	Trypsin\LysC
This study	AQKEEFF		2	449.75	17.66	897.42	18	24	7	3	20.3	Chymotrypsin
This study	AQKEEFFKTYVNLVNIIPAMKDVYWGK	Phospho [Y24]	4	828.68	37.62	3310.65	18	44	27	6	1.13	Trypsin\LysC
This study	FKTYVNLVNIIPAMKDVYW		4	579.34	16.69	2313.22	24	42	19	5	2.82	Chymotrypsin
This study	FKTYVNLVNIIPAMKDVYWGKD	Oxidation [M14] Phospho [T3 Y18]	4	698.32	10.40	2789.29	24	45	22	6	8.37	GluC
This study	KTYVNLVNIIPAMKDVYW	Oxidation [M13] Phospho [T2 Y3]	3	781.73	29.32	2342.08	25	42	18	5	0.42	Chymotrypsin
This study	KTYVNLVNIIPAMKDVYWGKD	Oxidation [M13] Phospho [T2]	3	856.09	24.51	2562.26	25	45	21	8	1.29	GluC
Vincent et al 2019	TYVNLVNIIPAMK		2	737.89	30.96	1473.76	26	38	13	0	3.03	Trypsin
Vincent et al 2019	TYVNLVNIIPAMK		2	737.94	32.06	1473.86	26	38	13	0	3.12	Trypsin
This study	TYVNLVNIIPAMK		2	738.40	32.75	1474.82	26	38	13	0	41.08	Trypsin\LysC
This study	TYVNLVNIIPAMK		2	738.92	28.61	1474.82	26	38	13	0	46.06	Trypsin\LysC
This study	TYVNLVNIIPAMK		2	738.41	31.29	1474.82	26	38	13	0	76.74	Trypsin\LysC
This study	TYVNLVNIIPAMK		2	738.42	28.58	1474.82	26	38	13	0	83.19	Trypsin\LysC
This study	TYVNLVNIIPAMKDVYWGKDVTQKNK	Oxidation [M12]	4	764.41	21.15	3052.61	26	51	26	3	6.16	Trypsin\LysC
This study	TYVNLVNIIPAMK	Oxidation [M12] Phospho [T1]	2	787.90	26.77	1570.78	26	38	13	0	2.12	Trypsin\LysC
This study	TYVNLVNIIPAMKDVYWGKDVTQKNKEEGYTHIVE	Phospho [T31]	4	1045.02	36.85	4174.05	26	60	35	8	4.14	GluC
This study	VNLVNIIPAMKDVY		2	795.39	30.58	1587.87	28	41	14	2	13.39	Chymotrypsin
This study	VNLVNIIPAMKDVY		2	794.94	29.95	1587.87	28	41	14	1	58.81	Chymotrypsin
This study	VNLVNIIPAMKDVYW	Oxidation [M10]	2	896.49	33.01	1789.94	28	42	15	3	19.3	Chymotrypsin
This study	VNLVNIIPAMKDVYW	Oxidation [M10]	2	895.97	32.88	1789.94	28	42	15	3	29.81	Chymotrypsin
This study	VNIIPAMKDVYWGK	Oxidation [M7] Phospho [Y11]	2	865.42	37.92	1728.83	31	44	14	4	2.68	Trypsin\LysC
Vincent et al 2019	DVYWGKDVTQK		2	668.35	18.61	1334.69	39	50	11	1	1.90	Trypsin
This study	DVTQKNKEE		2	545.77	12.76	1089.53	45	53	9	4	7.64	GluC
This study	EEGYTHIVEVTFESVETIQDYIIHPAHVGFGDVYR	Phospho [T11 S14 T17 Y34]	4	1093.25	37.66	4368.81	52	86	35	0	1.94	Trypsin\LysC
This study	SVETIQDYIIHPAHVGFGDVYR		3	839.75	25.85	2515.25	65	86	22	3	64.06	Trypsin\LysC
This study	IIHPAHVGF		3	331.20	15.48	989.54	73	81	9	0	12.13	Chymotrypsin
This study	IIHPAHVGF		3	330.86	15.80	989.54	73	81	9	0	22	Chymotrypsin
This study	IIHPAHVGFGDVY		3	475.58	19.97	1423.72	73	85	13	1	4.21	Chymotrypsin
This study	IIHPAHVGFGDVY		2	712.87	20.05	1423.72	73	85	13	1	9.07	Chymotrypsin
This study	RSFWEKLL		3	360.20	13.49	1077.60	86	93	8	5	2.66	Chymotrypsin
Vincent et al 2019	LLIFDYTPR		2	569.32	28.80	1136.62	92	100	9	0	2.12	Trypsin
This study	LLIFDYTPR		2	569.32	26.21	1136.62	92	100	9	1	47.9	Trypsin\LysC
Vincent et al 2019	LLIFDYTPRK		3	422.58	25.35	1264.73	92	101	10	1	1.74	Trypsin
This study	LLIFDYTPRK		4	317.19	13.85	1264.72	92	101	10	5	3.15	Trypsin\LysC
This study	LLIFDYTPRK		3	422.58	24.68	1264.72	92	101	10	1	17.19	Trypsin\LysC
This study	LLIFDYTPRK		3	422.58	22.12	1264.72	92	101	10	2	31.38	Trypsin\LysC
This study	LLIFDYTPRK		2	633.34	22.52	1264.72	92	101	10	2	40.05	Trypsin\LysC
This study	LLIFDYTPRK	Phospho [T7]	2	673.86	26.58	1344.68	92	101	10	2	0.27	Trypsin\LysC
This study	IFDYTPRK		3	347.20	14.82	1038.55	94	101	8	2	17.23	Trypsin\LysC

B

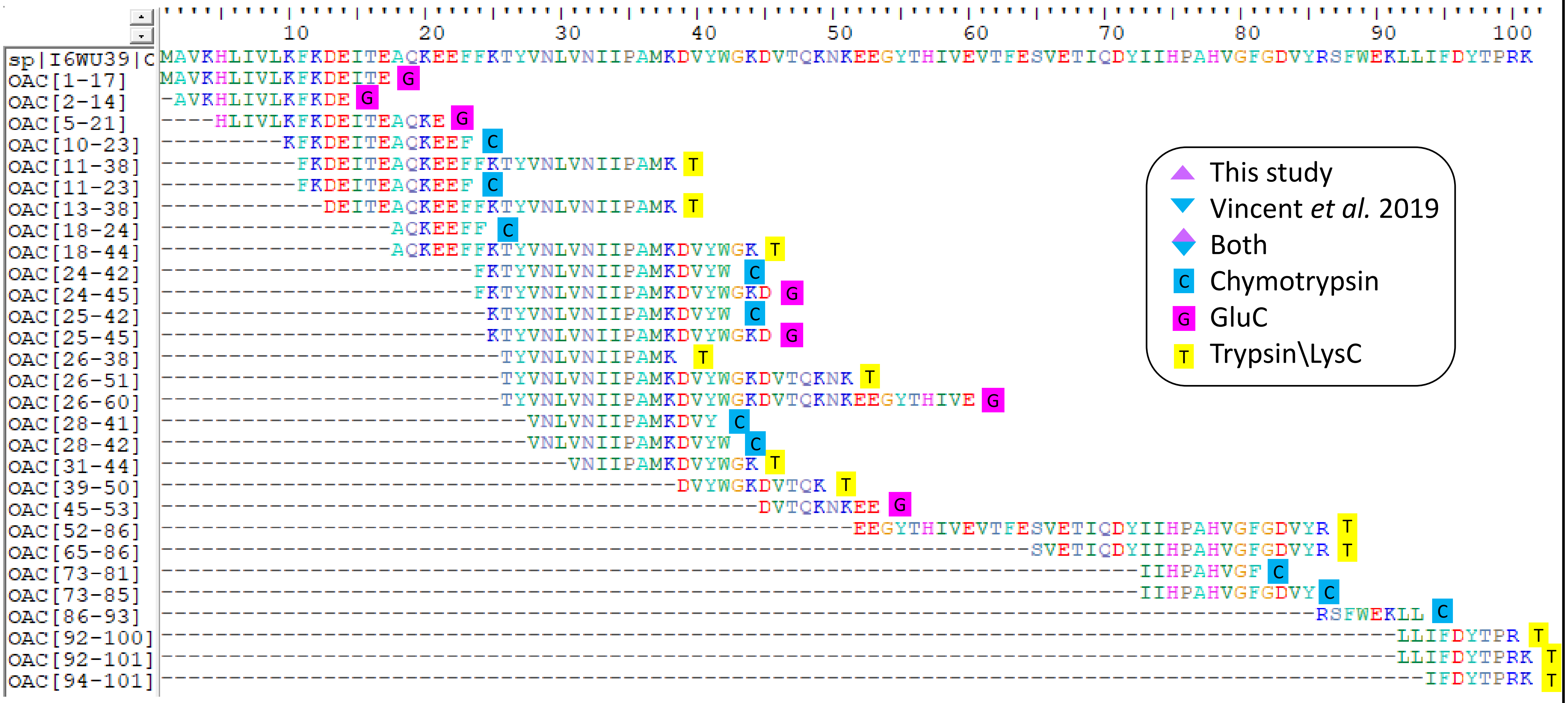

C

Vincent et al 2019: OAC 34% coverage (Trypsin only, max 2 missed cleavages)

This study: OAC 100% coverage (Trypsin/LysC 85%, GluC 57%, Chymotrypsin 53%, max 10 missed cleavages)

Supplementary Figure S9: **Cannabis enzymes involved in terpenoid (A) and cannabinoid (B) metabolisms.** KEGG pathway mapping was performed using E.C. numbers of cannabis enzymes. Enzymes identified in this study are highlighted in red. Enzyme full names are indicated in Supplementary Table S3.

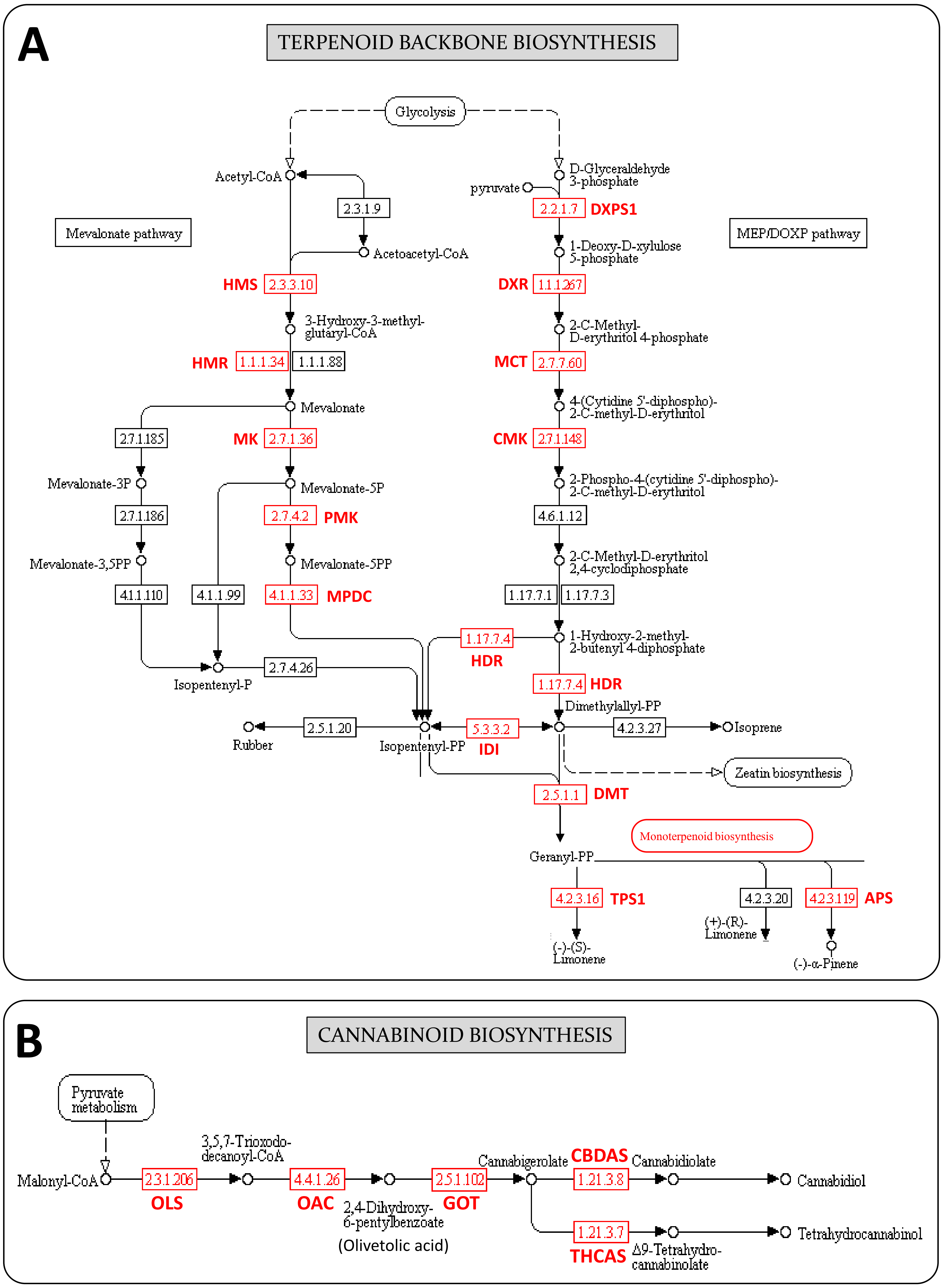

Supplement: Supplementary file 1 [file ijms-20-05630-s001.zip › Vincent_MC_protein digestion paper_Suppl Figures_2019-11-01.pdf]
